# Supplementary material for: Partial substitution of chemical fertilizer by organic fertilizer increases yield, quality and nitrogen utilization of Dioscorea polystachya
Source: PLoS One. 2024 Apr 11;19(4):e0301108. doi: 10.1371/journal.pone.0301108 (PMC11008863; doi:10.1371/journal.pone.0301108)
Supplement: Attachment — Submitted filename: PONE-D-23-21993 Rev.pdf [file pone.0301108.s003.pdf]

Partial Substitution of Chemical Fertilizer with Organic Fertilizer Increases Yield, Quality and Nitrogen Utilization of *Dioscorea polystachya*

Chao Sun [1], Hua Zheng [2], Qing Zhao [3], Yuxi Liu [4], Hai Liu [1]

(1. School of Management Science, Guizhou University of Finance and Economics, Guiyang 550025, China; 2. Guizhou Company, China National Tobacco Corporation, Guiyang 550001, China; 3. Academic Affairs Office, Guizhou University of Finance and Economics, Guiyang, Guizhou, China; 550025; 4. School of Literature, Guizhou University of Finance and Economics, Guiyang 550025, China)

Abstract: This field experiment aimed to investigate the effects of different ratios of organic and inorganic fertilizers with equal nitrogen application rates on the yield, quality, and nitrogen uptake efficiency of *Dioscorea polystachya* (yam). Six treatments were established, including a control without fertilizer (CK), sole application of chemical fertilizer (CF), sole application of organic fertilizer (OM), 25% organic fertilizer + 75% chemical fertilizer (25%OM + 75%CF), and 50% organic fertilizer + 50% chemical fertilizer (50%OM + 50%CF). The experiment followed a randomized complete block design with three replications. Various yield parameters, morphology, quality indicators, and nitrogen utilization were analyzed to assess the differences among treatments. The results indicated that all fertilizer treatments significantly increased the yield, morphology, quality indicators, and nitrogen utilization efficiency compared to the control. 25%OM + 75%CF achieved the highest yield of 31.96 t hm^{-2}, which was not significantly different from CF (30.18 t hm^{-2}). Although there were no significant differences in tuber length and marketable yield among the fertilizer treatments, 25%OM + 75%CF exhibited the highest values at 69.23 cm and 75.86%, respectively, representing a 3.14% and 1.57% increase compared to CF. Tuber thickness and fresh weight of 25%OM + 75%CF showed no significant differences from CF, while OM and 50%OM+50%CF exhibited varying degrees of reduction compared to CF. The total sugar, starch, crude protein, total amino acid, and ash contents of *D. polystachya* significantly increased in all fertilizer treatments compared to the control (except for ash content between CK and OM). When compared to CF, the substitution of different ratios of organic fertilizer for chemical fertilizer increased the total sugar, starch, crude protein, total amino acid, and ash contents to varying degrees. The treatment with 25%OM+75%CF exhibited the highest increases of 6.31%, 3.78%, 18.40%, 29.70%, and 10%, respectively. Nitrogen content in different plant parts followed the sequence of tuber > leaves > stems > aerial stem, with the highest nitrogen accumulation observed in 25%OM + 75%CF treatment. Nitrogen harvest index did not show significant differences among treatments, fluctuating between 0.69 and 0.74. The nitrogen apparent utilization efficiency was highest in 25%OM + 75%CF (9.89%), followed by CF (9.09%), both significantly higher than OM (5.32%) and 50%OM + 50%CF (6.69%). The nitrogen agronomic efficiency varied significantly among treatments, with 25%OM + 75%CF (33.93 kg kg^{-1}) being the highest, followed by CF (29.68 kg kg^{-1}), 50%OM + 50%CF (21.82 kg kg^{-1}), and OM (11.85 kg kg^{-1}). Nitrogen partial factor productivity was

highest in 25%OM + 75%CF treatment (76.37 kg kg^{-1}), followed by CF (72.11 kg kg^{-1}), both significantly higher than 50%OM + 50%CF (64.25 kg kg^{-1}) and OM (54.29 kg kg^{-1}), with OM exhibiting significantly lower values compared to other treatments. In conclusion, the combined application of organic and inorganic fertilizers can effectively enhance the yield, quality, and nitrogen utilization efficiency of *D. polystachya*. Particularly, the treatment with 25% organic fertilizer and 75% chemical fertilizer showed the most promising results.

Key words: *Dioscorea polystachya*; partial substitution of chemical fertilizer with organic fertilizer; yield; quality; nitrogen utilization

1. Introduction

Yam (*Dioscorea polystachya*) is a perennial herbal plant that holds significant economic value as an important crop in China (Fan et al., 2020). It is highly regarded for its nutritional and medicinal properties, serving as a crucial source of income for farmers (Li et al., 2023). However, the extensive growth cycle and high nutrient requirements of yam necessitate substantial fertilizer application. Unfortunately, in the pursuit of higher yields, farmers often resort to excessive use of chemical fertilizers, resulting in increased production costs and detrimental environmental effects (He et al., 2020; Dimkpa et al., 2020). Research has revealed that an excessive proportion of unused fertilizers remains in the soil, leading to issues such as soil compaction and salinization (Stavi et al., 2021). Additionally, these unused fertilizers can enter the atmosphere and water bodies through various pathways like ammonia volatilization, nitrification, denitrification, surface runoff, and leaching. As a result, this contributes to worsening atmospheric pollution and eutrophication of water bodies (Brentrup et al., 2004; Sun et al., 2012). Consequently, it becomes imperative to explore comprehensive agricultural development models and nutrient management strategies that effectively meet China's food demands while minimizing the strain on resources and the environment (Chivenge et al., 2022). This approach is crucial for achieving sustainable and "green" agricultural development in China (Liu et al., 2019).

Researchers have found that the use of organic fertilizer alone can lead to a significant decrease in crop yield. In low-input conditions, the application of organic fertilizer alone resulted in a 24% to 54% reduction in crop yield compared to the application of chemical fertilizer alone or the combined application of organic and inorganic fertilizers (Vanlauwe et al., 2011). Furthermore, a 6-year field trial in Sweden demonstrated that using manure as the sole fertilizer resulted in a 47% and 82% decrease in barley and potato yields, respectively (Torstensson et al., 2006). Many studies have highlighted the benefits of combining organic and chemical fertilizers, as this approach can boost crop yield and enhance soil quality. This combined application strategy holds great potential for increasing agricultural production, improving efficiency, and reducing fertilizer usage in China.

In Guizhou province, the predominant practice has been the reliance on chemical fertilizers, with limited use of organic fertilizers. To address this issue, this experiment aimed to determine the

optimal ratio of organic fertilizer substitution for chemical fertilizer in the yam cultivation area of Guizhou. The study assessed the effects of different proportions of organic fertilizer as a replacement for chemical fertilizer on yam yield, quality, morphology, and nitrogen utilization efficiency. The findings of this study aim to provide valuable guidance on reducing chemical fertilizer usage, promoting the appropriate application of organic fertilizer, and effectively enhancing yam yield in the Guizhou yam planting region.

2. Materials and methods

2.1. Overview of the test site

The experimental site was situated in the University Town of Huaxi District, Guiyang City, Guizhou Province, China (26° 22′N, 106° 38′E). This location falls within the hilly area of central Guizhou Province, characterized by an average altitude of 1030 m. The region experiences a subtropical monsoon climate, with an average annual temperature of 15.3℃ and approximately 1350 hours of sunshine per year. The frost-free period spans around 270 days, and annual rainfall typically ranges between 1100-1200mm.

The soil type at the experimental field is classified as yellow soil, with Triassic limestone and sand shale residue as the parent material. In terms of its basic physical and chemical properties, the topsoil (0 ~ 20 cm) exhibits the following characteristics: Organic matter content of 15.41 g/kg, total nitrogen accumulation of 0.82 g/kg, total phosphorus content of 0.41 g/kg, total potassium content of 11.89 g/kg, alkali hydrolyzed nitrogen concentration of 75.18 mg/kg, available phosphorus level of 16.8 mg/kg, available potassium concentration of 109.6 mg/kg, and a pH value of 5.74.

2.2. Test material

1) Yam variety: The test variety used in this study was Anshun Yam, a locally adapted variety obtained from the cultivation of wild yam plants in the region. This variety has undergone long-term domestication and natural selection. Seeds of Anshun Yam were acquired from Huaxi District Seed Company, located in Guiyang City, Guizhou Province, China.

2) Fertilizers: Fertilizers for the experiment included organic earthworm manure, which had a nutrient composition of N-P_2O_5-K_2O=2.01-2.41-1.03 and an organic matter content of 36%. This organic earthworm manure was purchased from Guizhou Jilong Ecological Technology Co., LTD. In addition, synthetic fertilizers were used, including urea (N 46%), compound fertilizer (N-P_2O_5-K_2O =15-15-15), superphosphate (P_2O_5 16%), potassium sulfate (K_2O 50%), and diammonium hydrogen phosphate (N 18%, P_2O_5 46%). All of these synthetic fertilizers were procured from a local agricultural materials company.

Comentado [A1]: What was the input to obtain the vermicompost?

2.3. Experimental design

The experiment was conducted over a two-year period, from 2020 to 2021, to evaluate the effects of different treatments on yam cultivation. Five treatments were established:

1) Control (CK): No fertilization was applied;

2) Chemical fertilizer (CF): Single application of conventional chemical fertilizer;

3) Organic fertilizer (OM): Single application of organic fertilizer;

4) 25%OM+75%CF: 25% organic fertilizer + 75% chemical fertilizer;

5) 50%OM+50%CF: 50% organic fertilizer + 50% chemical fertilizer.

A randomized block design was employed, and the experiment was replicated three times. The experimental plots had an area of 27 m^2 (7.5 m × 3.6 m) and were divided into 15 plots. Each plot consisted of six rows, with a row spacing of 30 cm × 60 cm. A total of 150 yam plants (equivalent to 55 560 plants/hm^2) were planted in each plot, with a 0.3 m walkway separating the plots. Protective rows were established around the experimental plots to minimize edge effects.

Comentado [A2]: Why only 3 repetitions?

Comentado [A3]: Considering the experimental design, the number of treatments and replicates, the degrees of freedom of error are few. A serious experiment considers at least 12 degrees of freedom from error.

In both 2020 and 2021, the sowing of yam seeds took place on March 12 using the branch trench sowing method. The trenches were dug to a depth of 15 cm. Prior to sowing, a base fertilizer was applied, and the soil was then bedded after sowing. Once the yam seedlings emerged in an orderly manner, they were set up, and additional fertilizer was applied when the yams were fully grown. Conventional field management practices were followed.

For Treatment 2, which involved local conventional fertilization, the specific types and amounts of fertilizers applied per 667m^2 were as follows: 150 kg of compound fertilizer as the base fertilizer, 50 kg of potassium sulfate as a topdressing, and 30 kg of diammonium hydrogen phosphate as a topdressing. The amounts of nitrogen, phosphorus, and potassium applied per hectare were equal for each fertilization treatment. This means that per hectare, 418.5 kg of N, 544.5 kg of P_2O_5, and 712.5 kg of K_2O were applied. The application amount of organic fertilizer was determined based on its nitrogen content, and any deficiency in phosphorus and potassium fertilizers was supplemented using potassium sulfate and superphosphate, respectively. Specifically, the compound fertilizer, organic fertilizer, and superphosphate were all applied as base fertilizers, while urea, potassium sulfate, and diammonium hydrogen phosphate were split with 60% applied as base fertilizer and the remaining 40% applied as topdressing.

2.4. Measuring items and methods

Soil properties: The basic physical and chemical properties of the topsoil were assessed using standard conventional methods in the field test.

Yam yield: The yam harvest period was determined based on the actual yield of the experimental plots. The average yield from three replicate plots was calculated to obtain the overall yield.

Tuber morphology: Ten random yam plants were selected from each plot to measure tuber morphology indices, including tuber length and tuber thickness.

Yam quality: Fresh and intact yam tubers of medium length were chosen from each plot to

determine quality indices. The total ash content, soluble sugar content (using the anthrone colorimetric method), starch content (using the colorimetric method), and soluble protein content (using the Coomas bright blue colorimetric method) were determined.

Nutrient content: For nutrient analysis, five randomly selected yam plants were collected from each plot. The stems, leaves, rhizomes, and tubers were weighed individually. The green parts were heated at 105℃ for 30 minutes to deactivate enzymes, and then dried at 65℃ to constant weight. The dry weight was recorded, and the samples were crushed for further analysis. After a deboiling process using concentrated sulfuric acid and hydrogen peroxide, the total nitrogen content (using the Kjeldahl method), total phosphorus content (using the vanadium-molybdenum yellow colorimetric method), and total potassium content (using a flame photometer) were determined for each plant part.

Relevant calculation formulas:

Plant nitrogen accumulation (kg hm^{-2}) = dry matter mass per plant × number of plants per hectare × nitrogen content of plants.

Nitrogen harvest index (NHI) = nitrogen accumulation of tuber / nitrogen accumulation of the whole plant.

Apparent nitrogen utilization ratio (%) = (nitrogen accumulation of plants treated with N fertilization－nitrogen accumulation of plants treated without N fertilization) / N fertilization amount × 100.

Nitrogen agronomic efficiency (kg/kg) = (yield of plants treated with N fertilization － yield of plants treated without N fertilization) / amount of nitrogen applied.

Nitrogen partial productivity (kg/kg) = yield / amount of nitrogen applied.

2.5. Data processing and analysis

All data were analyzed using one-way ANOVA in SAS 9.4 software (SAS Institute Inc., Cary, NC, USA). The analysis included the determination of yam yield, morphological indicators, total sugar, starch, crude protein, total amino acid, moisture, and ash content, as well as N accumulation in tuber, stem, leaf, and bulbil, and N use efficiency. Correlations between all above indicators in different treatments were also analyzed. The least significant difference (LSD) was employed for multiple comparisons between treatment means. The mean difference is considered significant when $P < 0.05$.

Comentado [A4]: No correlation results are presented.

3. Results and analysis

3.1. Yield

As shown in Figure 1, compared to the control treatment (CK), all fertilization treatments significantly increased yam yield ($P < 0.05$). The effect of different treatment varied. Among them, 25%OM + 75%CF had the highest yield, reaching 31.96 t hm^{-2}, which was significantly higher than OM (22.72 t hm^{-2}) and 50%OM + 50%CF (26.89 t hm^{-2}), but showed no significant difference from

CF (30.18 t hm^{-2}). The yield increase achieved by OM was the weakest, significantly lower than that of other fertilizer treatments.

3.2. Morphological indexes

As shown in Table 2, fertilization significantly improved the morphological indexes of yam, although the effects varied among different fertilization treatments. 25%OM+75%CF resulted in the highest values for tuber length, tuber diameter, tuber fresh weight, and commercial rate, which were 69.23 cm, 5.51 cm, 1.15 kg, and 75.86%, respectively. There were no significant differences in tuber length and commodity rate among the different treatments, with values ranging from 63.48 cm to 69.23 cm and from 74.12% to 75.86%, respectively. However, the tuber diameter of OM was significantly lower than that of CF and 25%OM+75%CF (5.48 cm and 5.51 cm, respectively). The tuber fresh weight showed significant differences among different treatments, except for the non-significant difference between CF (1.11 kg) and 25%OM+75%CF (1.15 kg). Significant differences were observed among the other treatments ($P < 0.05$).

Table 2 Effect of partial substitution of chemical fertilizer with organic fertilizer on morphological indicators of yam

Treatment	Tuber length /cm	Tuber thickness /cm	Tuber fresh weight /kg	Commodity rate /%
CK	56.78± 1.08c	4.66± 0.13c	0.68± 0.07d	60.87± 1.03b
CF	67.12± 1.35a	5.48± 0.14a	1.11± 0.12a	74.69± 1.77a
OM	63.48±0.81 ab	4.87± 0.07b	0.80± 0.06c	75.16± 2.38a
25%OM+75%CF	69.23± 2.17a	5.51± 0.26a	1.15± 0.13a	75.86± 2.65a
50%OM+50%CF	65.31±1.79 ab	5.03± 0.11ab	0.91± 0.05b	74.12± 1.14a

Note: Different letters in the same column indicate significant level of difference P < 0.05

3.3. Quality

Application of fertilizer has a significant impact on the quality of yam. Fertilization led to a significant increase in the contents of total sugar, starch, crude protein, total amino acid, and ash in yam (except for OM), while significantly reducing the water content of yam ($P < 0.05$). The contents of total sugar, starch, water, and ash in each fertilizer treatment varied within the ranges of 12.21%～12.98%, 15.62%～16.21%, 75.44%～76.95%, and 0.66%～0.77%, respectively. However, no significant differences were observed among the different treatments. Compared to CF, partial substitution of chemical fertilizer with organic fertilizer significantly increased the contents of crude protein and total amino acid in yam, with the treatment of 25%OM+75%CF showing the highest increases at 18.40% and 29.70%, respectively (Table 3).

Table 3 Effect of partial substitution of chemical fertilizer with organic fertilizer on yam quality

Treatment	Total sugar /%	Starch /%	Crude protein /%	Total amino acids /%	Moisture /%	Ash /%
CK	10.43± 0.28b	14.01± 0.08b	2.81± 0.07c	1.74±0.03 c	81.23± 1.68a	0.61± 0.02b
CF	12.21± 0.31a	15.62± 0.11a	3.37± 0.11b	2.02± 0.05b	76.95± 1.94b	0.70± 0.01a

OM	12.69± 0.24a	15.66± 0.18a	3.91± 0.16a	2.59±0.06 a	75.18± 1.79b	0.66± 0.04ab
25%OM+75%CF	12.98± 0.33a	16.21± 0.13a	3.99± 0.15a	2.62±0.09 a	75.44± 2.14b	0.77±0.03 a
50%OM+50%CF	12.77±0.29 a	15.78± 0.17a	3.86± 0.19a	2.55± 0.02a	76.25± 1.81b	0.71± 0.05a

Note: Different letters in the same column indicate significant difference at $P < 0.05$ level.

3.4. Nitrogen accumulation

The distribution of nitrogen accumulation in different parts of yam follows the pattern of tuber > leaf > stem > bulbil, with proportions ranging from 68.81% to 73.76%, 15.74% to 17.73%, 8.35% to 8.72%, and 2.16% to 4.95%, respectively (Fig.2). In terms of total nitrogen accumulation, fertilization significantly increased the nitrogen accumulation in yam ($P < 0.05$). 25%OM+75%CF had the highest nitrogen accumulation at 156.91 kg hm^{-2}, followed by CF (153.56 kg hm^{-2}), with no significant difference between them. However, both were significantly higher than that of OM (137.78 kg hm^{-2}) and 50%OM+50%CF (143.49 kg hm^{-2}).

Comentado [A5]: Maintain uniformity in the literals. In lower case as in figure 1.

3.5. Nitrogen utilization

As shown in Table 4, the nitrogen harvest index of the control treatment (CK) was the highest, reaching 0.74. However, there were no significant differences between CK and other treatments, and the nitrogen harvest index (NHI) ranged between 0.69 and 0.74. 25%OM+75%CF had the highest nitrogen apparent utilization rate at 9.89%, followed by CF(9.09%). Both of these values were significantly higher than 50%OM+50%CF (6.69%) and OM (5.32%). Significant differences were observed in nitrogen agronomic efficiency among the different treatments. 25%OM+75%CF had the highest nitrogen agronomic efficiency at 33.93 kg kg^{-1}, followed by CF (29.68 kg kg^{-1}). 50%OM+50%CF and OM had the lowest nitrogen agronomic efficiency of 21.82 kg kg^{-1} and 11.85 kg kg^{-1}, respectively. The nitrogen partial productivity was the highest for 25%OM+75%CF at 76.37 kg kg^{-1}, followed by CF (72.11 kg kg^{-1}). There were no significant differences between these two treatments, but both were significantly higher than 50%OM+50%CF (64.25 kg kg^{-1}) and OM (54.29 kg kg^{-1}).

Table 4 Effect of organic and chemical fertilizer application on nitrogen use efficiency of yam (mean ± standard deviation)

Treatment	NHI	Apparent utilization rate /%	Agronomic efficiency /(kg kg^{-1})	Partial productivity /(kg kg^{-1})
CK	0.74±0.01 a	—	—	—
CF	0.70±0.03 a	9.09±0.44 a	29.68±1.79 b	72.11±2.44 a
OM	0.69±0.02 a	5.32±0.51 b	11.85±0.46 d	54.29±2.19 c
25%OM+75%CF	0.70±0.01 a	9.89±0.76 a	33.93±1.35 a	76.37±3.76 a
50%OM+50%CF	0.70±0.02 a	6.69±0.48 b	21.82±1.08 c	64.25±5.48 b

Note: Different letters in the same column indicate significant difference at $P < 0.05$ level.

4. Conclusion and discussion

Comentado [A6]: Further discussion related to the characteristics and effects of the organic material applied is required.

Comentado [A7]: Practically only nitrogen is discussed, what about the other essential elements?

In comparison to the conventional fertilizer treatment (CF), the application of organic fertilizer instead of 25% of the fertilizer significantly increased yam yield. However, when organic fertilizer replaced 50% of the fertilizer, the yield was reduced (Suja et al., 2014). Previous studies have

indicated that when all nitrogen is provided by organic fertilizer, it can lead to a decrease in yield (Geng et al., 2019). Therefore, it is crucial to pay attention to the combined application ratio of organic and chemical fertilizers. A high proportion of organic fertilizer can be detrimental to crop production and result in yield reduction. Under the condition of equal nitrogen supply, replacing 50% of fertilizer nitrogen with organic fertilizer can lead to slow nutrient release and lower ammonium and nitrate nitrogen content in the soil. As yam's nitrogen demand increases during the later stages of growth, it cannot meet the necessary growth requirements, ultimately resulting in a significant decline in yield. This finding is consistent with previous research (Divito et al., 2019). As a result, two consecutive fertilization experiments have demonstrated the feasibility of replacing a portion of fertilizer with organic fertilizer in yam cultivation in Guizhou Province. Maintaining a ratio of 25% organic fertilizer nitrogen to fertilizer nitrogen allows for yams to maintain high tuber length, crude and fresh weight, and commercial rate, ensuring a stable and high yield.

Proper nitrogen application has been shown to improve the nitrogen absorption intensity and distribution rate of yam grains, and the combined application of organic fertilizer and chemical fertilizer can increase the nitrogen accumulation in yam plants and significantly enhance the nitrogen content in stems (KV et al., 2019; Hgaza et al., 2012). Our study confirmed that the nitrogen accumulation and distribution ratio in yam stems were significantly higher than in other organs, which aligns with previous research findings (Srivastava et al., 2010). Under isonitrogen fertilization conditions, a 25% ratio of organic fertilizer nitrogen to fertilizer nitrogen resulted in significantly higher total nitrogen accumulation in plants and nitrogen accumulation in seeds compared to conventional fertilizer treatment. Wu et al. (2010) also demonstrated, using 'N labeling technology, that the combined application of organic and inorganic fertilizer could enhance nitrogen accumulation in grains of both vegetative and reproductive organs of winter wheat compared to single application of organic or inorganic fertilizer. The optimal accumulation and distribution of crop dry matter play a crucial role in achieving high-quality and high-yield yam production. The combined application of organic fertilizer and chemical fertilizer is beneficial for optimizing water and nutrient conditions, promoting overall dry matter accumulation throughout the crop's growth period, and facilitating the transfer of dry matter to the stems (Liu et al., 2022; Büyük et al., 2022). However, as the ratio of organic fertilizer nitrogen to fertilizer nitrogen increases, both total nitrogen accumulation in plants and nitrogen accumulation in grains gradually decrease, consistent with previous studies (Qi et al., 2021; Li et al., 2021; Wang et al., 2023; Raza et al., 2021). Furthermore, it has been observed that as the proportion of organic fertilizer replacing chemical fertilizer increases, the organic matter content also increases. However, the mineralization process of organic matter is relatively slow, leading to a slower release of nutrients. Consequently, the nitrogen mineralized from organic fertilizer may be insufficient to meet the plant's nitrogen uptake requirements, resulting in inadequate absorption of available nitrogen and ultimately lower nitrogen accumulation in the stems (Moe et al., 2020; Xiao et al., 2022).

Comentado [A8]: Any organic fertilizer?

Comentado [A9]: In the list of references it appears as 2014.

Comentado [A10]: In the list of references it appears as 2020.

Low nitrogen utilization rate is a prominent challenge in crop production in China. Various indicators, such as nitrogen harvest index, nitrogen apparent utilization rate, agronomic efficiency, and partial productivity, can be used to measure nitrogen efficiency. In this study, significant differences were observed in both the agronomic efficiency and partial productivity of nitrogen fertilizer. These findings indicate an imbalance in nitrogen, phosphorus, and potassium fertilization or excessive fertilizer application in the current fertilization system in this region. Therefore, optimizing the fertilizer structure is necessary. Research has demonstrated that, under a certain nitrogen application level, reducing chemical fertilizer application and increasing organic fertilizer application can greatly improve the fertilizer utilization efficiency of yam. However, it is important to note that the ratio of organic fertilizer to fertilizer nitrogen should be optimized rather than simply aiming for a higher ratio (Pouya et al., 2022). Li et al. (2018) found that the highest nitrogen fertilizer agronomic use efficiency of wheat was achieved when organic fertilizer replaced 20% of the chemical nitrogen fertilizer. In our study, we observed a similar trend, where nitrogen fertilizer efficiency increased initially and then decreased with an increase in the proportion of organic fertilizer nitrogen replacing chemical fertilizer nitrogen. The highest nitrogen use efficiency of yam was achieved when the proportion of organic fertilizer nitrogen replacing chemical fertilizer nitrogen was 25%. This result aligns with previous research findings (Ma et al., 2013). Application of organic fertilizer can enhance soil physical, chemical, and biological properties, promote root growth, facilitate nitrogen absorption and utilization by crops, improve crop yield, and ultimately increase nitrogen efficiency.

Acknowledgements

This work received funding from the Guizhou University of Finance and Economics 2021 introduction of talent scientific research start-up project (Grant No. 2021YJ051).

Reference

Brentrup F, Küsters J, Kuhlmann H, et al. Environmental impact assessment of agricultural production systems using the life cycle assessment methodology: I. Theoretical concept of a LCA method tailored to crop production[J]. European Journal of Agronomy, 2004, 20(3): 247-264.

Büyük G, Bayram C A, İnan M, et al. The effect of organic and inorganic fertilizers on plant nutrient content and agronomic performance of stevia[J]. Journal of Plant Nutrition, 2022, 45(15): 2303-2314.

Chivenge P, Zingore S, Ezui K S, et al. Progress in research on site-specific nutrient management for smallholder farmers in sub-Saharan Africa[J]. Field Crops Research, 2022, 281: 108503.

Dimkpa C O, Fugice J, Singh U, et al. Development of fertilizers for enhanced nitrogen use efficiency–Trends and perspectives[J]. Science of the Total Environment, 2020, 731: 139113.

Divito G A, Sadras V O. How do phosphorus, potassium and sulphur affect plant growth and biological nitrogen fixation in crop and pasture legumes? A meta-analysis[J]. Field Crops Research, 2014, 156: 161-171.

Comentado [A11]: In the text it appears as 2019.

Fan D, Zhong H, Hu B, et al. Agro-ecological suitability assessment of Chinese Medicinal Yam under future climate change[J]. Environmental geochemistry and health, 2020, 42: 987-1000.

Geng Y, Cao G, Wang L, et al. Effects of equal chemical fertilizer substitutions with organic manure on yield, dry matter, and nitrogen uptake of spring maize and soil nitrogen distribution[J]. PloS one, 2019, 14(7): e0219512.

He R, Shao C, Shi R, et al. Development trend and driving factors of agricultural chemical fertilizer efficiency in China[J]. Sustainability, 2020, 12(11): 4607. https://doi.org/10.3390/su12114607

Hgaza V K, Diby L N, Oberson A, et al. Nitrogen use by yam as affected by mineral fertilizer application[J]. Agronomy Journal, 2012, 104(6): 1558-1568.

KV H, Oberson A, Kiba I D, et al. The nitrogen nutrition of yam (Dioscorea spp)[J]. Journal of Plant Nutrition, 2020, 43(1): 64-78.

Li F F, Zhu H Y, Duan J S, et al. Effect of combined application of inorganic and organic fertilizer on wheat yield and fertilizer use efficiency in wheat following rice[J]. Journal of Triticum Crops, 2018, 38(5): 593-599. (in Chinese)

Li K, Wang C, Li X, et al. Long-term effect of integrated fertilization on maize yield and soil fertility in a calcaric fluvisol[J]. Archives of Agronomy and Soil Science, 2021, 67(10): 1400-1410.

Li Y, Ji S, Xu T, et al. Chinese yam (*Dioscorea*): Nutritional value, beneficial effects, and food and pharmaceutical applications[J]. Trends in Food Science & Technology, 2023, 134: 29-40.

Liu M, Linna C, Ma S, et al. Biochar combined with organic and inorganic fertilizers promoted the rapeseed nutrient uptake and improved the purple soil quality[J]. Frontiers in Nutrition, 2022, 9: 997151.

Liu P, He J, Li H, et al. Effect of straw retention on crop yield, soil properties, water use efficiency and greenhouse gas emission in China: A meta-analysis[J]. International Journal of Plant Production, 2019, 13: 347-367.

Ma T T, Cai F, Ding C Y, et al. Biological effects of special bio-organic fertilizer on yam[J]. Journal of Nanjing Agricultural University, 2013, 36(4): 83-90. (in Chinese)

Moe K, Htwe A Z, Dien D C, et al. Effects of organic fertilizer applied using the estimated mineralizable nitrogen method on nitrogen uptake, growth characteristics, yield, and yield components of Genkitsukushi rice (Oryza sativa)[J]. Journal of plant nutrition, 2020, 43(10): 1400-1417.

Pouya N, Hgaza V K, Kiba D I, et al. Water yam (Dioscorea alata L.) growth and tuber yield as affected by rotation and fertilization regimes across an environmental gradient in West Africa[J]. Agronomy, 2022, 12(4): 792.

Comentado [A12]: In the text it appears as 2019.

Qi Y, Jiang F, Zhou R, et al. Effects of reduced nitrogen with bio-organic fertilizer on soil properties, yield and quality of non-heading Chinese cabbage[J]. Agronomy, 2021, 11(11): 2196.

Raza S T, Wu J, Ali Z, et al. Differential effects of organic amendments on maize biomass and nutrient availability in upland calcareous soil[J]. Atmosphere, 2021, 12(8): 1034.

Srivastava A, Dagbenonbakin G D, Gaiser T. Effect of fertilization on yam (Dioscorea rotundata) biomass production[J]. Journal of plant nutrition, 2010, 33(7): 1056-1065.

Stavi I, Thevs N, Priori S. Soil salinity and sodicity in drylands: A review of causes, effects, monitoring, and restoration measures[J]. Frontiers in Environmental Science, 2021: 330.

Suja G, Sreekumar J. Implications of organic management on yield, tuber quality and soil health in yams in the humid tropics[J]. International Journal of Plant Production, 2014, 8(3): 291-310.

Sun B, Zhang L, Yang L, et al. Agricultural non-point source pollution in China: causes and mitigation measures[J]. Ambio, 2012, 41: 370-379.

Torstensson G, Aronsson H, Bergström L. Nutrient use efficiencies and leaching of organic and conventional cropping systems in Sweden[J]. Agronomy Journal, 2006, 98(3): 603-615.

Vanlauwe B, Kihara J, Chivenge P, et al. Agronomic use efficiency of N fertilizer in maize-based systems in sub-Saharan Africa within the context of integrated soil fertility management[J]. Plant and soil, 2011, 339: 35-50.

Wang J, Zhang X, Yuan M, et al. Effects of Partial Replacement of Nitrogen Fertilizer with Organic Fertilizer on Rice Growth, Nitrogen Utilization Efficiency and Soil Properties in the Yangtze River Basin[J]. Life, 2023, 13(3): 624.

Wu C L, Shen Q R, Xia Z Y, et al. Mechanisms for the increased utilization of fertilizer N under integrated use of inorganic and organic fertilizers in a winter wheat-rice rotation system. I. Fate of fertilizer 15N during winter wheat growing stages[J]. Acta Pedologica Sinica, 2010, 47(5): 905-912.

Xiao X, Li J, Lyu J, et al. Chemical fertilizer reduction combined with bio-organic fertilizers increases cauliflower yield via regulation of soil biochemical properties and bacterial communities in Northwest China[J]. Frontiers in Microbiology, 2022, 13: 922149.
