# Supplementary material for: Partial substitution of chemical fertilizer by organic fertilizer increases yield, quality and nitrogen utilization of Dioscorea polystachya
Source: PLoS One. 2024 Apr 11;19(4):e0301108. doi: 10.1371/journal.pone.0301108 (PMC11008863; doi:10.1371/journal.pone.0301108)
Supplement: Attachment — Submitted filename: PONE-D-23-21993_R1_reviewer-1.pdf [file pone.0301108.s005.pdf]

PLOS ONE

Partial Substitution of Chemical Fertilizer with Organic Fertilizer Increases Yield, Quality and Nitrogen Utilization of Dioscorea polystachya

--Manuscript Draft--

**Manuscript Number:**	PONE-D-23-21993R1
**Article Type:**	Research Article
**Full Title:**	Partial Substitution of Chemical Fertilizer with Organic Fertilizer Increases Yield, Quality and Nitrogen Utilization of Dioscorea polystachya
**Short Title:**	Partial Substitution of Chemical Fertilizer with Organic Fertilizer Increases Yam Yield and Quality
**Corresponding Author:**	Hai Liu<br>Guizhou University of Finance and Economics<br>Guiyang, Guizhou CHINA
**Keywords:**	Dioscorea polystachya; partial substitution of chemical fertilizer with organic fertilizer; yield; Quality; nitrogen utilization
**Abstract:**	This field experiment aimed to investigate the effects of different ratios of organic and inorganic fertilizers with equal nitrogen application rates on the yield, quality, and nitrogen uptake efficiency of Dioscorea polystachya (yam). Six treatments were established, including a control without fertilizer (CK), sole application of chemical fertilizer (CF), sole application of organic fertilizer (OM), 25% organic fertilizer + 75% chemical fertilizer (25%OM + 75%CF), and 50% organic fertilizer + 50% chemical fertilizer (50%OM + 50%CF). The experiment followed a randomized complete block design with three replications. Various yield parameters, morphology, quality indicators, and nitrogen utilization were analyzed to assess the differences among treatments. The results indicated that all fertilizer treatments significantly increased the yield, morphology, quality indicators, and nitrogen utilization efficiency compared to the control. 25%OM + 75%CF achieved the highest yield of 31.96 t hm-2, which was not significantly different from CF (30.18 t hm-2). Although there were no significant differences in tuber length and marketable yield among the fertilizer treatments, 25%OM + 75%CF exhibited the highest values at 69.23 cm and 75.86%, respectively, representing a 3.14% and 1.57% increase compared to CF. Tuber thickness and fresh weight of 25%OM + 75%CF showed no significant differences from CF, while OM and 50%OM+50%CF exhibited varying degrees of reduction compared to CF. The total sugar, starch, crude protein, total amino acid, and ash contents of D. polystachya significantly increased in all fertilizer treatments compared to the control (except for ash content between CK and OM). When compared to CF, the substitution of different ratios of organic fertilizer for chemical fertilizer increased the total sugar, starch, crude protein, total amino acid, and ash contents to varying degrees. The treatment with 25%OM+75%CF exhibited the highest increases of 6.31%, 3.78%, 18.40%, 29.70%, and 10%, respectively. Nitrogen content in different plant parts followed the sequence of tuber > leaves > stems > aerial stem, with the highest nitrogen accumulation observed in 25%OM + 75%CF treatment. Nitrogen harvest index did not show significant differences among treatments, fluctuating between 0.69 and 0.74. The nitrogen apparent utilization efficiency was highest in 25%OM + 75%CF (9.89%), followed by CF (9.09%), both significantly higher than OM (5.32%) and 50%OM + 50%CF (6.69%). The nitrogen agronomic efficiency varied significantly among treatments, with 25%OM + 75%CF (33.93 kg kg-1) being the highest, followed by CF (29.68 kg kg-1), 50%OM + 50%CF (21.82 kg kg-1), and OM (11.85 kg kg-1). Nitrogen partial factor productivity was highest in 25%OM + 75%CF treatment (76.37 kg kg-1), followed by CF (72.11 kg kg-1), both significantly higher than 50%OM + 50%CF (64.25 kg kg-1) and OM (54.29 kg kg-1), with OM exhibiting significantly lower values compared to other treatments. In conclusion, the combined application of organic and inorganic fertilizers can effectively enhance the yield, quality, and nitrogen utilization efficiency of D. polystachya. Particularly, the treatment with 25% organic fertilizer and 75% chemical fertilizer showed the most promising results.
**Order of Authors:**	Chao Sun
	Hua Zheng

Powered by Editorial Manager® and ProduXion Manager® from Aries Systems Corporation

	Qing Zhao
	Yuxi Liu
	Hai Liu
**Response to Reviewers:**	Thank you very much for your positive comments on our manuscript entitled “Partial Substitution of Chemical Fertilizer with Organic Fertilizer Increases Yield, Quality and Nitrogen Utilization of Dioscorea polystachya” (PONE-D-23-21993). All the comments are valuable and helpful for improving our paper. We substantially revised our old version based on the reviewer’s comments. Thereafter, the manuscript was revised and edited by a native speaker and we hope the revised manuscript can meet the requirements. In this thoroughly revised version, changes in our manuscript within the document were highlighted by using red-colored text. Point-by-point responses to the reviewers are included, and the line number based on the attached file named “Revised Manuscript with track changes”.
**Additional Information:**	
**Question**	**Response**
**Financial Disclosure**<br><br>Enter a financial disclosure statement that describes the sources of funding for the work included in this submission. Review the submission guidelines for detailed requirements. View published research articles from *PLOS ONE* for specific examples.<br><br>This statement is required for submission and **will appear in the published article** if the submission is accepted. Please make sure it is accurate.<br><br>**Unfunded studies**<br>Enter: *The author(s) received no specific funding for this work.*<br><br>**Funded studies**<br>Enter a statement with the following details:<br>• Initials of the authors who received each award<br>• Grant numbers awarded to each author<br>• The full name of each funder<br>• URL of each funder website<br>• Did the sponsors or funders play any role in the study design, data collection and analysis, decision to publish, or preparation of the manuscript?<br>• **NO** - Include this sentence at the end of your statement: *The funders had no role in study design, data collection and analysis, decision to publish, or preparation of the manuscript.*<br>• **YES** - Specify the role(s) played.	This research was funded by the Guizhou University of Finance and Economics 2021 introduction of talent scientific research start-up project (Grant No. 2021YJ051). The funders had no role in study design, data collection and analysis, decision to publish, or preparation of the manuscript.

Powered by Editorial Manager® and ProduXion Manager® from Aries Systems Corporation

* typeset	
**Competing Interests**<br><br>Use the instructions below to enter a competing interest statement for this submission. On behalf of all authors, disclose any competing interests that could be perceived to bias this work—acknowledging all financial support and any other relevant financial or non-financial competing interests.<br><br>This statement is required for submission and **will appear in the published article** if the submission is accepted. Please make sure it is accurate and that any funding sources listed in your Funding Information later in the submission form are also declared in your Financial Disclosure statement.<br><br>View published research articles from *PLOS ONE* for specific examples.<br><br>**NO authors have competing interests**<br><br>Enter: *The authors have declared that no competing interests exist.*<br><br>**Authors with competing interests**<br><br>Enter competing interest details beginning with this statement:<br><br>*I have read the journal's policy and the authors of this manuscript have the following competing interests: [insert competing interests here]*<br><br>* typeset	The authors have declared that no competing interests exist.
**Ethics Statement**<br><br>Enter an ethics statement for this submission. This statement is required if the study involved:<br><br>• Human participants<br>• Human specimens or tissue	N/A

• Vertebrate animals or cephalopods<br>• Vertebrate embryos or tissues<br>• Field research<br><br>Write "N/A" if the submission does not require an ethics statement.<br><br>General guidance is provided below. Consult the submission guidelines for detailed instructions. **Make sure that all information entered here is included in the Methods section of the manuscript.**	

Powered by Editorial Manager® and ProduXion Manager® from Aries Systems Corporation

**Format for specific study types**<br><br>**Human Subject Research (involving human participants and/or tissue)**<br>• Give the name of the institutional review board or ethics committee that approved the study<br>• Include the approval number and/or a statement indicating approval of this research<br>• Indicate the form of consent obtained (written/oral) or the reason that consent was not obtained (e.g. the data were analyzed anonymously)<br><br>**Animal Research (involving vertebrate animals, embryos or tissues)**<br>• Provide the name of the Institutional Animal Care and Use Committee (IACUC) or other relevant ethics board that reviewed the study protocol, and indicate whether they approved this research or granted a formal waiver of ethical approval<br>• Include an approval number if one was obtained<br>• If the study involved *non-human primates*, add *additional details* about animal welfare and steps taken to ameliorate suffering<br>• If anesthesia, euthanasia, or any kind of animal sacrifice is part of the study, include briefly which substances and/or methods were applied<br><br>**Field Research**<br><br>Include the following details if this study involves the collection of plant, animal, or other materials from a natural setting:<br>• Field permit number<br>• Name of the institution or relevant body that granted permission	
**Data Availability**<br><br>Authors are required to make all data underlying the findings described fully available, without restriction, and from the time of publication. PLOS allows rare exceptions to address legal and ethical concerns. See the PLOS Data Policy and FAQ for detailed information.	Yes - all data are fully available without restriction

A Data Availability Statement describing where the data can be found is required at submission. Your answers to this question constitute the Data Availability Statement and **will be published in the article**, if accepted.<br><br>**Important:** Stating 'data available on request from the author' is not sufficient. If your data are only available upon request, select 'No' for the first question and explain your exceptional situation in the text box.<br><br>Do the authors confirm that all data underlying the findings described in their manuscript are fully available without restriction?	
**Describe where the data may be found in full sentences. If you are copying our sample text, replace any instances of XXX with the appropriate details.**<br><br>• If the data are **held or will be held in a public repository**, include URLs, accession numbers or DOIs. If this information will only be available after acceptance, indicate this by ticking the box below. For example: *All XXX files are available from the XXX database (accession number(s) XXX, XXX.*).<br>• If the data are all contained **within the manuscript and/or Supporting Information files**, enter the following: *All relevant data are within the manuscript and its Supporting Information files.*<br>• If neither of these applies but you are able to provide **details of access elsewhere**, with or without limitations, please do so. For example:<br><br>*Data cannot be shared publicly because of [XXX]. Data are available from the XXX Institutional Data Access / Ethics Committee (contact via XXX) for researchers who meet the criteria for access to confidential data.*<br><br>*The data underlying the results presented in the study are available from (include the name of the third party*	All relevant data are within the manuscript and its Supporting Information files

Powered by Editorial Manager® and ProduXion Manager® from Aries Systems Corporation

and contact information or URL).<br>• This text is appropriate if the data are owned by a third party and authors do not have permission to share the data.<br><br>* typeset	
Additional data availability information:	

Manuscript
Click here to access/download;Manuscript;Revised Manuscript.docx

Partial Substitution of Chemical Fertilizer with Organic Fertilizer Increases Yield, Quality and Nitrogen Utilization of *Dioscorea polystachya*

Chao Sun[1], Hua Zheng[2], Qing Zhao[3], Yuxi Liu[4], Hai Liu[1*]

[1]School of Management Science and Engineering, Guizhou University of Finance and Economics, Guiyang, Guizhou, China

[2]Guizhou Tobacco Company, China National Tobacco Corporation, Guiyang, Guizhou, China

[3]Academic Affairs Office, Guizhou University of Finance and Economics, Guiyang, Guizhou, China

[4]School of Literature, Guizhou University of Finance and Economics, Guiyang, Guizhou, China)

* Corresponding author

E-mail: liuhai200321@163.com

Abstract

This field experiment aimed to investigate the effects of different ratios of organic and inorganic fertilizers with equal nitrogen application rates on the yield, quality, and nitrogen uptake efficiency of *Dioscorea polystachya* (yam). Six treatments were set, including a control without fertilizer (CK), sole application of chemical fertilizer (CF), sole application of organic fertilizer (OM), 25% organic fertilizer + 75% chemical fertilizer (25%OM + 75%CF), 50% organic fertilizer + 50% chemical fertilizer (50%OM + 50%CF), and 75% organic fertilizer + 25% chemical fertilizer (75%OM + 25%CF). The experiment followed a randomized complete block design with three replications. Various yield parameters, morphology, quality indicators, and nitrogen utilization were analyzed to assess the differences among treatments. The results indicated that all fertilizer treatments significantly increased the yield, morphology, quality indicators, and nitrogen utilization efficiency compared to the control. 25%OM + 75%CF achieved the highest yield of 31.96 t hm^{-2}, which was not significantly different from CF (30.18 t hm^{-2}). Although there were no significant differences in tuber length and marketable yield among the fertilizer treatments, 25%OM + 75%CF exhibited the highest values at 69.23 cm and 75.86%, respectively, representing a 3.14% and 1.57% increase compared to CF. Tuber thickness and fresh weight of 25%OM + 75%CF showed no significant differences from CF, while OM and 50%OM+50%CF exhibited varying degrees of reduction compared to CF. The total sugar, starch, crude protein, total amino acid, and ash contents of *D. polystachya* significantly increased in all fertilizer treatments compared to the control (except for ash content between CK and OM). When compared to CF, the substitution of different ratios of organic fertilizer for chemical fertilizer increased the total sugar, starch, crude protein, total amino acid, and ash contents to varying degrees. The treatment with 25%OM+75%CF exhibited the highest increases of 6.31%, 3.78%, 18.40%, 29.70%, and 10%, respectively. Nitrogen content in different plant parts followed the sequence of tuber > leaves > stems > aerial stem, with the highest nitrogen accumulation observed in 25%OM + 75%CF treatment. Nitrogen harvest index did not show significant differences among

treatments, fluctuating between 0.69 and 0.74. The nitrogen apparent utilization efficiency was highest in 25%OM + 75%CF (9.89%), followed by CF (9.09%), both significantly higher than OM (5.32%) and 50%OM + 50%CF (6.69%). The nitrogen agronomic efficiency varied significantly among treatments, with 25%OM + 75%CF (33.93 kg kg^{-1}) being the highest, followed by CF (29.68 kg kg^{-1}), 50%OM + 50%CF (21.82 kg kg^{-1}), and OM (11.85 kg kg^{-1}). Nitrogen partial factor productivity was highest in 25%OM + 75%CF treatment (76.37 kg kg^{-1}), followed by CF (72.11 kg kg^{-1}), both significantly higher than 50%OM + 50%CF (64.25 kg kg^{-1}) and OM (54.29 kg kg^{-1}), with OM exhibiting significantly lower values compared to other treatments. In conclusion, the combined application of organic and inorganic fertilizers can effectively enhance the yield, quality, and nitrogen utilization efficiency of *D. polystachya*. Particularly, the treatment with 25% organic fertilizer and 75% chemical fertilizer showed the most promising results.

Key words: *Dioscorea polystachya*; partial substitution of chemical fertilizer with organic fertilizer; yield; quality; nitrogen utilization

Introduction

Yam (*Dioscorea polystachya*) is a perennial herbal plant that holds significant economic value as an important crop in China [1]. It is highly regarded for its nutritional and medicinal properties, serving as a crucial source of income for farmers [2]. However, the extensive growth cycle and high nutrient requirements of yam necessitate substantial fertilizer application. Unfortunately, in the pursuit of higher yields, farmers often resort to excessive use of chemical fertilizers, resulting in increased production costs and detrimental environmental effects [3, 4]. Research has revealed that an excessive proportion of unused fertilizers remains in the soil, leading to issues such as soil compaction and salinization [5]. Additionally, these unused fertilizers can enter the atmosphere and water bodies through various pathways like ammonia volatilization, nitrification, denitrification, surface runoff, and leaching. As a result, this contributes to worsening atmospheric pollution and eutrophication of water bodies [6, 7]. Consequently, it becomes imperative to explore comprehensive agricultural development models and nutrient management strategies that effectively meet China's food demands while minimizing the strain on resources and the environment [8]. This approach is crucial for achieving sustainable and "green" agricultural development in China [9].

Researchers have found that the use of organic fertilizer alone can lead to a significant decrease in crop yield. In low-input conditions, the application of organic fertilizer alone resulted in a 24% to 54% reduction in crop yield compared to the application of chemical fertilizer alone or the combined application of organic and inorganic fertilizers [10]. Furthermore, a 6-year field trial in Sweden demonstrated that using manure as the sole fertilizer resulted in a 47% and 82% decrease in barley and potato yields, respectively [11]. Many studies have highlighted the benefits of combining organic and chemical fertilizers, as this approach can boost crop yield and enhance soil quality. This

combined application strategy holds great potential for increasing agricultural production, improving efficiency, and reducing fertilizer usage in China.

In Guizhou province, the predominant practice has been the reliance on chemical fertilizers, with limited use of organic fertilizers. The study assessed the effects of different proportions of organic fertilizer as a replacement for chemical fertilizer on yam yield, quality, morphology, and nitrogen utilization efficiency. The findings of this study aim to provide valuable guidance on reducing chemical fertilizer usage, promoting the appropriate application of organic fertilizer, and effectively enhancing yam yield in the Guizhou yam planting region.

Materials and methods

Overview of the test site

The experimental site was situated in the University Town of Huaxi District, Guiyang City, Guizhou Province, China (26° 22′N, 106° 38′E). This location falls within the hilly area of central Guizhou Province, characterized by an average altitude of 1030 m. The region experiences a subtropical monsoon climate, with an average annual temperature of 15.3℃ and approximately 1350 hours of sunshine per year. The frost-free period spans around 270 days, and annual rainfall typically ranges between 1100-1200 mm.

The experimental yellow soil (Eutric Regosol, UAO Soil Taxonomic System) was sampled from 0～20 cm soil layer prior to the experiment and then measured (analysis methods listed below). The soil derived from Triassic limestone and sand shale deposits, and had a sandy loam texture with pH 5.74 and contained 15.41 g/kg of organic matter (OM), 0.82 g/kg of total N (TN), 0.41 g/kg of total P (TP), 11.89 g/kg of total K (TK), 75.18 mg/kg of 1N NaOH-hydrolyzed N (AN), 16.8 mg/kg of Olsen P (AP), and 109.6 mg/kg of 1N CH_3COONH_4-exchangeable K (AK).

Test material

1) Yam variety: The test variety used in this study was Anshun Yam, a locally adapted variety obtained from the cultivation of wild yam plants in the region. This variety has undergone long-term domestication and natural selection. Seeds of Anshun Yam were acquired from Huaxi District Seed Company, located in Guiyang City, Guizhou Province, China.

2) Fertilizers: The organic fertilizer vermicompost, obtained by digesting decomposed cow dung with earthworms, had a nutrient composition of N-P_2O_5-K_2O=2.01-2.41-1.03 and an organic matter content of 36%. The vermicompost was purchased from Guizhou Jilong Ecological Technology Co., LTD. In addition, synthetic fertilizers were used, including urea (N 46%), compound fertilizer (N-P_2O_5-K_2O =15-15-15), superphosphate (P_2O_5 16%), potassium sulfate (K_2O 50%), and diammonium hydrogen phosphate (N 18%, P_2O_5 46%). All of these synthetic fertilizers were procured from a local

agricultural materials company.

Experimental design

The experiment was conducted over a two-year period, from 2020 to 2021, to evaluate the effects of different treatments on yam cultivation. Six treatments were established:

1) Control (CK): No fertilization was applied;

2) Chemical fertilizer (CF): Sole application of conventional chemical fertilizer;

3) Organic fertilizer (OM): Sole application of organic fertilizer;

4) 25%OM+75%CF: 25% organic fertilizer + 75% chemical fertilizer;

5) 50%OM+50%CF: 50% organic fertilizer + 50% chemical fertilizer;

6) 75%OM+25%CF: 75% organic fertilizer + 25% chemical fertilizer.

The experiment was design in a randomized complete block design with three replicates. The experimental plot size was 7.5 m × 3.6 m. Each plot consisted of six rows, with a row spacing of 30 cm × 60 cm. A total of 180 yam plants (equivalent to 55 560 plants/hm^2) were planted in each plot, with a 0.3 m walkway separating the plots. Protective rows were established around the experimental plots to minimize edge effects.

In both 2020 and 2021, yam setts were planted on March 12 using the trench planting method (trench depth 15 cm). Prior to planting, a basal application of fertilizers was conducted, followed by ridging after planting (ridge height 15 cm). Staking was done before sprouting, and then trailing operations were continuously conducted by trailing newly sprouted yam vines to the stakes. Top dressing was applied approximately two months after planted. The field management practices were in accordance with local high-quality cultivation technical regulations. All fertilizing treatments were of the same application rates of N 418.5 kg/ha, P_2O_5 544.5 kg/ha, and K_2O 712.5 kg/ha. CF was the local conventional fertilizer treatment, with 2250 kg/ha compound fertilizer as base fertilizer, 750.0 kg/ha potassium sulfate plus 450.0 kg/ha ammonium dihydrogen phosphate as topdressing fertilizer. For the treatments combining organic and inorganic fertilizers, the organic fertilizer was applied in terms of nitrogen content, and any deficiency was supplemented with urea, calcium superphosphate, and potassium sulfate. Organic fertilizer and calcium superphosphate were exclusively used for basal application, while 60% of urea, potassium sulfate, and ammonium dihydrogen phosphate were used for basal application, and the remaining 40% was applied as topdressing. The fertilizer type and application amount were presented in Table 1.

Table 1. Fertilization schemes of different treatments (kg/ha)

Treatments	Base fertilizer	Topdressing fertilizer

	Compound fertilizer	Urea	Calcium superphosphate	Potassium sulfate	Ammonium dihydrogen phosphate	Organic fertilizer	Urea	Potassium sulfate	Ammonium dihydrogen phosphate
CK	0	0	0	0	0	0	0	0	0
CF	2250	0	0	0	0	0	0	750.0	450.0
OM	0	0	267.2	597.6	0	20820	0	398.4	0
25%OM+75%CF	0	409.4	2619	790.7	0	5205	273.0	527.1	0
50%OM+50%CF	0	273.0	1835	726.3	0	10410	182.0	484.2	0
75%OM+25%CF	0	136.5	1051	662.0	0	15615	91.05	441.3	0

Measuring items and methods

The yam was harvested during Nov. 21- Nov. 30, plot yield was weighed individually, soil (0～20 cm in depth) and yam tuber samples were collected through five-point sampling method in each plot separately. The measuring items and methods were listed below.

Soil properties: The soil samples collected from each plot were air-dried for determining the following indicators. The soil pH (soil:water =1:10) was measured with a pH metre (FE20, Mettler Toledo Co., Ltd., Shanghai, China), and OM was detected by the $K_2Cr_2O_7$ oxidation method. After digested with H_2SO_4-H_2O_2, the soil TN, TP, and TK in the digests were respectively determined by methods of Kjeldahl distillation, molybdenum blue colorimetry and flame photospectrometry [12]. For AN determination, the soil samples were evenly mixed with $FeSO_4 \cdot 7H_2O$-Ag_2SO_4 and added with 1 N NaOH solution (soil: solution = 1:5, weight: volume), after incubated at 40 °C for 24 h, HCl titration was employed to determine NH3 collected in 2% H_3BO_3 solution. 0.5 M $NaHCO_3$ (soil:water =1:10) was prepared for AP extraction, which was then measured using the colorimetric molybdenum-blue method. AK was extracted with 1 M NH4Ac and determined with a flame photometer (Shanghai Analysis Instrument Co., Ltd., China).

Yam yield: After sampling we harvested yam tubers per plot separately. The average yield of three replicate plots of each treatment was weighed and calculated to obtain the overall yield.

Tuber morphology: Ten random yam plants were selected from each plot to measure tuber morphology indices, including tuber length and tuber thickness.

Yam quality: Fresh and intact yam tubers of medium length were chosen from each plot to determine quality indices. The total ash content, soluble sugar content, starch content, and soluble protein content were determined respectively by the colorimetric analysis method [13].

Nutrient content: For nutrient analysis, five randomly selected yam plants were collected from each plot. The stems, leaves, rhizomes, and tubers were weighed individually. The green parts were heated at 105℃ for 30 minutes to deactivate enzymes, and then dried at 65℃ to constant weight. The dry weight was recorded, and the samples were crushed for further analysis. After a deboiling process

using concentrated sulfuric acid and hydrogen peroxide, the total nitrogen content (Kjeldahl method), total phosphorus content (vanadium-molybdenum yellow colorimetric method), and total potassium content (flame photometer) were determined for each plant part [14].

Relevant calculation formulas:

Plant nitrogen accumulation (kg hm^{-2}) = dry matter mass per plant × number of plants per hectare × nitrogen content of plants.

Nitrogen harvest index (NHI) = nitrogen accumulation of tuber / nitrogen accumulation of the whole plant.

Apparent nitrogen utilization ratio (%) = (nitrogen accumulation of plants treated with N fertilization－nitrogen accumulation of plants treated without N fertilization) / N fertilization amount × 100.

Nitrogen agronomic efficiency (kg/kg) = (yield of plants treated with N fertilization － yield of plants treated without N fertilization) / amount of nitrogen applied.

Nitrogen partial productivity (kg/kg) = yield / amount of nitrogen applied.

Data processing and analysis

All data were analyzed using one-way ANOVA in SAS 9.4 software (SAS Institute Inc., Cary, NC, USA). The analysis included the determination of yam yield, morphological indicators, total sugar, starch, crude protein, total amino acid, moisture, and ash content, as well as N accumulation in tuber, stem, leaf, and bulbil, and N use efficiency. The least significant difference (LSD) was employed for multiple comparisons between treatment means. The mean difference is considered significant when $P < 0.05$.

Results and analysis

Yield

As shown in Fig 1, compared to the control treatment (CK), all fertilization treatments significantly increased yam yield ($P < 0.05$). The effect of different treatment varied. Among them, 25%OM + 75%CF had the highest yield, reaching 31.96 t ha^{-1}, which was significantly higher than OM (22.72 t ha^{-1}), 50%OM + 50%CF (26.89 t ha^{-1}) and 75%OM + 25%CF (24.38 t ha^{-1}), but showed no significant difference from CF (30.18 t ha^{-1}). The yield increase achieved by OM was the weakest, significantly lower than that of other fertilizer treatments.

Morphological indexes

As shown in Table 2, fertilization significantly improved the morphological indexes of yam, although the effects varied among different fertilization treatments. 25%OM+75%CF resulted in the

highest values for tuber length, tuber diameter, tuber fresh weight, and commercial rate, which were 69.23 cm, 5.51 cm, 1.15 kg, and 75.86%, respectively. There were no significant differences in tuber length and commodity rate among the different treatments, with values ranging from 63.48 cm to 69.23 cm and from 74.12% to 75.86%, respectively. However, the tuber diameter of OM was significantly lower than that of CF and 25%OM+75%CF (5.48 cm and 5.51 cm, respectively). The tuber fresh weight showed significant differences among different treatments, except for the non-significant difference of CF (1.11 kg) with 25%OM+75%CF (1.15 kg), as well as that of 75%OM+25%CF (0.82 kg) with OM (0.80 kg) and 50%OM+50%CF (0.91 kg). Significant differences were observed among the other treatments ($P < 0.05$).

Table 2. Effect of partial substitution of chemical fertilizer with organic fertilizer on morphological indicators of yam

Treatment	Tuber length /cm	Tuber thickness /cm	Tuber fresh weight /kg	Commodity rate /%
CK	56.78± 1.08c	4.66± 0.13c	0.68± 0.07d	60.87± 1.03b
CF	67.12± 1.35a	5.48± 0.14a	1.11± 0.12a	74.69± 1.77a
OM	63.48±0.81 ab	4.87± 0.07b	0.80± 0.06c	75.16± 2.38a
25%OM+75%CF	69.23± 2.17a	5.51± 0.26a	1.15± 0.13a	75.86± 2.65a
50%OM+50%CF	65.31±1.79 ab	5.03± 0.11ab	0.91± 0.05b	74.12± 1.14a
75%OM+25%CF	64.63±1.21 ab	4.92±0.09 ab	0.82±0.08 bc	74.31±0.82 a

Note: Different letters in the same column indicate significant level of difference P < 0.05

Quality

Application of fertilizer has a significant impact on the quality of yam. Fertilization led to a significant increase in the contents of total sugar, starch, crude protein, total amino acid, and ash in yam (except for OM), while significantly reducing the water content of yam ($P < 0.05$). The contents of total sugar, starch, water, and ash in each fertilizer treatment varied within the ranges of 12.21%～12.98%, 15.62%～16.21%, 75.43%～76.95%, and 0.66%～0.77%, respectively. However, no significant differences were observed among the different treatments. Compared to CF, partial substitution of chemical fertilizer with organic fertilizer significantly increased the contents of crude protein and total amino acid in yam, with the treatment of 25%OM+75%CF showing the highest increases at 18.40% and 29.70%, respectively (Table 3).

Table 3. Effect of partial substitution of chemical fertilizer with organic fertilizer on yam quality

Treatment	Total sugar /%	Starch /%	Crude protein /%	Total amino acids /%	Moisture /%	Ash /%
CK	10.43± 0.28b	14.01± 0.08b	2.81± 0.07c	1.74±0.03 c	81.23± 1.68a	0.61± 0.02b
CF	12.21± 0.31a	15.62± 0.11a	3.37± 0.11b	2.02± 0.05b	76.95± 1.94b	0.70± 0.01a
OM	12.69± 0.24a	15.66± 0.18a	3.91± 0.16a	2.59±0.06 a	75.18± 1.79b	0.66± 0.04ab
25%OM+75%CF	12.98± 0.33a	16.21± 0.13a	3.99± 0.15a	2.62±0.09 a	75.44± 2.14b	0.77±0.03 a

50%OM+50%CF	12.77±0.29 a	15.78± 0.17a	3.86± 0.19a	2.55± 0.02a	76.25± 1.81b	0.71± 0.05a
75%OM+25%CF	12.75±0.17 a	15.71± 0.14a	3.87± 0.12a	2.52± 0.04a	75.43± 1.26b	0.67± 0.04ab

Note: Different letters in the same column indicate significant difference at $P < 0.05$ level.

Nitrogen accumulation

The distribution of nitrogen accumulation in different parts of yam follows the pattern of tuber > leaf > stem > bulbil, with proportions ranging from 68.81% to 73.76%, 15.74% to 17.73%, 8.35% to 8.72%, and 2.16% to 4.95%, respectively (Fig 2). In terms of total nitrogen accumulation, fertilization significantly increased the nitrogen accumulation in yam ($P < 0.05$). 25%OM+75%CF had the highest nitrogen accumulation at 156.91 kg ha^{-1}, followed by CF (153.56 kg ha^{-1}), with no significant difference between them. However, both were significantly higher than that of OM (137.78 kg ha^{-1}), 50%OM+50%CF (143.49 kg ha^{-1}), and 75%OM+25%CF (143.78 kg ha^{-1}).

Nitrogen utilization

As shown in Table 4, the nitrogen harvest index of the control treatment (CK) was the highest, reaching 0.74. However, there were no significant differences between CK and other treatments, and the nitrogen harvest index (NHI) ranged between 0.69 and 0.74. 25%OM+75%CF had the highest nitrogen apparent utilization rate at 9.89%, followed by CF(9.09%). Both of these values were significantly higher than 75%OM+25%CF (6.76%), 50%OM+50%CF (6.69%) and OM (5.32%). Significant differences were observed in nitrogen agronomic efficiency among the different treatments. 25%OM+75%CF had the highest nitrogen agronomic efficiency at 33.93 kg kg^{-1}, followed by CF (29.68 kg kg^{-1}). 75%OM+25%CF and OM had the lowest nitrogen agronomic efficiency of 15.82 kg kg^{-1} and 11.85 kg kg^{-1}, respectively. The nitrogen partial productivity was the highest for 25%OM+75%CF at 76.37 kg kg^{-1}, followed by CF (72.11 kg kg^{-1}). There were no significant differences between these two treatments, but both were significantly higher than 50%OM+50%CF (64.25 kg kg^{-1}), 75%OM+25%CF (58.26 kg kg^{-1}) and OM (54.29 kg kg^{-1}).

Table 4. Effect of organic and chemical fertilizer application on nitrogen use efficiency of yam(mean ± standard deviation)

Treatment	NHI	Apparent utilization rate /%	Agronomic efficiency /(kg kg^{-1})	Partial productivity /(kg kg^{-1})
CK	0.74±0.01 a	—	—	—
CF	0.70±0.03 a	9.09±0.44 a	29.68±1.79 b	72.11±2.44 a
OM	0.69±0.02 a	5.32±0.51 b	11.85±0.46 e	54.29±2.19 c
25%OM+75%CF	0.70±0.01 a	9.89±0.76 a	33.93±1.35 a	76.37±3.76 a
50%OM+50%CF	0.70±0.02 a	6.69±0.48 b	21.82±1.08 c	64.25±5.48 b
75%OM+25%CF	0.70±0.01 a	6.76±0.44 b	15.82±1.37 d	58.26±4.47 c

Note: Different letters in the same column indicate significant difference at $P < 0.05$ level.

Discussion

It is worth underscoring that achieving the right balance between organic and chemical fertilizers

is pivotal in optimizing nitrogen absorption, crop growth, and yield. This equilibrium not only enhances the understanding of nutrient dynamics in yam cultivation but also offers valuable insights for sustainable and high-yield agricultural practices in a broader context. In comparison to the conventional fertilizer treatment (CF), the substitution of 25% of chemical fertilizer with organic fertilizer resulted in a significant increase in yam yield. However, when organic fertilizer replaced 50% of the chemical fertilizer, there was a notable reduction in yield [15]. Prior research has suggested that an exclusive reliance on organic fertilizer as the sole nitrogen source can lead to yield decreases [16]. Consequently, it is imperative to consider the proportional blend of organic and chemical fertilizers. Under conditions of equal nitrogen supply, replacing 50% of the fertilizer's nitrogen with organic fertilizer can lead to a slower release of nutrients and reduced ammonium and nitrate nitrogen content in the soil. As yam's nitrogen requirements intensify during the later growth stages, these demands cannot be adequately met, ultimately resulting in a substantial decline in yield. This observation aligns with prior studies [17]. Our two-year fertilization experiment illustrates the viability of replacing a portion of chemical fertilizer with organic fertilizer in yam cultivation in Guizhou Province. We conclude that maintaining a 25% ratio of organic fertilizer nitrogen to fertilizer nitrogen preserves high tuber length, crude and fresh weight, and commercial rate in yam crops, ensuring a consistent and substantial yield.

Applying nitrogen correctly has been demonstrated to be highly effective in enhancing nitrogen absorption intensity and distribution rates in yam grains. The combined application of organic and chemical fertilizers has proven to increase nitrogen accumulation in yam plants and notably elevate the nitrogen content in the stems [18-19]. Our study revealed that the nitrogen accumulation and distribution ratio in yam stems significantly surpassed that of other plant parts, consistent with earlier research findings [20]. Under conditions of isonitrogen fertilization, replacing 25% of chemical fertilizers with organic fertilizer resulted in significantly greater total nitrogen accumulation throughout the entire plant and in the yam tubers compared to the conventional fertilizer treatment. Wu et al. [21], utilizing 'N labeling technology, also illustrated that combining organic and inorganic fertilizers could enhance nitrogen accumulation in grains within both vegetative and reproductive plant organs of winter wheat, surpassing the effects of single applications of organic or inorganic fertilizers. Optimal accumulation and distribution of crop dry matter play a pivotal role in achieving high-quality and high-yield yam production. The combined application of organic and chemical fertilizers proves beneficial in optimizing water and nutrient conditions, promoting overall dry matter accumulation throughout the crop's growth cycle, and facilitating the translocation of dry matter to the stems [22-23].

Nonetheless, as the proportion of organic fertilizer nitrogen relative to fertilizer nitrogen increases, both the total nitrogen accumulation in plants and grains gradually decreases, in accordance with prior studies [24-27]. Moreover, it has been observed that with the growing proportion of organic fertilizer substituting chemical fertilizer, there is an increase in organic matter content. However, the

mineralization process of organic matter is relatively slow, leading to a delayed release of nutrients. Consequently, the nitrogen mineralized from organic fertilizer may prove insufficient to meet the plant's nitrogen uptake requirements, resulting in inadequate absorption of available nitrogen and ultimately lower nitrogen accumulation in the stems [28-29].

The challenge of low nitrogen utilization rates remains a prominent issue in Chinese crop production. Various indicators, including nitrogen harvest index, nitrogen apparent utilization rate, agronomic efficiency, and partial productivity, serve to gauge nitrogen efficiency. Within the scope of this study, significant differences were observed concerning both agronomic efficiency and partial productivity of nitrogen fertilizer. These findings signify an imbalance in the fertilization system in the region, stemming from the application of nitrogen, phosphorus, and potassium or possibly excessive fertilizer use. Consequently, optimizing the fertilizer composition becomes a necessity. Research has indicated that, under specific nitrogen application levels, reducing chemical fertilizer application while increasing organic fertilizer usage can substantially enhance yam's fertilizer utilization efficiency. It is crucial to emphasize that the optimization should target the organic fertilizer-to-fertilizer nitrogen ratio rather than merely seeking a higher ratio [30]. Li et al. [31] discovered that the peak nitrogen fertilizer agronomic use efficiency in wheat was attained when organic fertilizer replaced 20% of the chemical nitrogen fertilizer. In our study, we observed a similar pattern, with nitrogen fertilizer efficiency initially increasing and then decreasing as the proportion of organic fertilizer nitrogen replacing chemical fertilizer nitrogen rose. The highest nitrogen use efficiency for yams was achieved when organic fertilizer nitrogen replaced 25% of chemical fertilizer nitrogen. This outcome aligns with previous research results [32]. These findings underscore the importance of a nuanced approach to nitrogen management in yam cultivation, seeking the optimal balance between organic and chemical fertilizers to maximize nitrogen utilization efficiency and overall crop productivity. The results of this study provide valuable insights for sustainable agricultural practices and contribute to the broader understanding of nutrient management in crop production.

Conclusion

Partial substituting chemical fertilizers with organic fertilizers performed differently as the substitution ratio varied in increasing yam yield, quality and nitrogen utilization of *D. polystachya*. Replacing 25% chemical fertilizers with organic fertilizer had comparable or even better performance than totally applying chemical fertilizers, specifically, the yam yield, tuber length, thickness, fresh weight, commodity rate, contents of total sugar, starch, moisture and ash, nitrogen accumulation amount, as well as nigrogen harvest index, apparent utilization rate and partial productivity didn’t vary significantly between CF and 25%OM+75%CF treatments, while the latter one significantly increased the contents of crude protein and total amino acids, and the agronomic efficiency. At the current stage, taking into the economic benefits for farmers, it is not practical to solely use organic

fertilizers without applying chemical fertilizers.

Acknowledgements

Many thanks to Dr. Jianguo Huang at Southwest University and anonymous reviewers for their valuable comments on this manuscript.

Author Contributions

Data curation: Chao Sun, Yuxi Liu.

Software: Qing Zhao.

Writing – original draft: Chao Sun, Hai Liu.

Writing – review & editing: Hai Liu.

References

1. Fan D, Zhong H, Hu B, et al. Agro-ecological suitability assessment of Chinese Medicinal Yam under future climate change. Environmental Geochemistry and Health. 2020; 42: 987-1000.
2. Li Y, Ji S, Xu T, et al. Chinese yam (*Dioscorea*): Nutritional value, beneficial effects, and food and pharmaceutical applications. Trends in Food Science & Technology. 2023; 134: 29-40.
3. He R, Shao C, Shi R, et al. Development trend and driving factors of agricultural chemical fertilizer efficiency in China. Sustainability. 2020, 12(11): 4607. https://doi.org/10.3390/su12114607.
4. Dimkpa CO, Fugice J, Singh U, et al. Development of fertilizers for enhanced nitrogen use efficiency–Trends and perspectives. Science of the Total Environment. 2020; 731: 139113.
5. Stavi I, Thevs N, Priori S. Soil salinity and sodicity in drylands: A review of causes, effects, monitoring, and restoration measures. Frontiers in Environmental Science. 2021: 330.
6. Brentrup F, Küsters J, Kuhlmann H, et al. Environmental impact assessment of agricultural production systems using the life cycle assessment methodology: I. Theoretical concept of a LCA method tailored to crop production. European Journal of Agronomy. 2004; 20(3): 247-264.
7. Sun B, Zhang L, Yang L, et al. Agricultural non-point source pollution in China: causes and mitigation measures. Ambio. 2012; 41: 370-379.
8. Chivenge P, Zingore S, Ezui K S, et al. Progress in research on site-specific nutrient management for smallholder farmers in sub-Saharan Africa. Field Crops Research. 2022; 281: 108503.
9. Liu P, He J, Li H, et al. Effect of straw retention on crop yield, soil properties, water use efficiency and greenhouse gas emission in China: A meta-analysis. International Journal of Plant Production. 2019; 13: 347-367.

10. Vanlauwe B, Kihara J, Chivenge P, et al. Agronomic use efficiency of N fertilizer in maize-based systems in sub-Saharan Africa within the context of integrated soil fertility management. Plant and soil. 2011; 339: 35-50.

11. Torstensson G, Aronsson H, Bergström L. Nutrient use efficiencies and leaching of organic and conventional cropping systems in Sweden. Agronomy Journal. 2006; 98(3): 603-615.

12. Pansu M. Handbook of soil analysis. Heidelberg: Springer; 2006.

13. Leng F, Sun S, Jing Y, et al. A rapid and sensitive method for determination of trace amounts of glucose by anthrone-sulfuric acid method. Bulg Chem Commun. 2016; 48(1): 109-113.

14. Motsara MR. Guide to laboratory establishment for plant nutrient analysis. Jodhpur: Scientific Publishers; 2015.

15. Suja G, Sreekumar J. Implications of organic management on yield, tuber quality and soil health in yams in the humid tropics. International Journal of Plant Production. 2014; 8(3): 291-310.

16. Geng Y, Cao G, Wang L, et al. Effects of equal chemical fertilizer substitutions with organic manure on yield, dry matter, and nitrogen uptake of spring maize and soil nitrogen distribution. PloS one, 2019; 14(7): e0219512.

17. Divito GA, Sadras VO. How do phosphorus, potassium and sulphur affect plant growth and biological nitrogen fixation in crop and pasture legumes? A meta-analysis. Field Crops Research. 2014; 156: 161-171.

18. KV H, Oberson A, Kiba I D, et al. The nitrogen nutrition of yam (*Dioscorea* spp). Journal of Plant Nutrition. 2020; 43(1): 64-78.

19. Hgaza VK, Diby LN, Oberson A, et al. Nitrogen use by yam as affected by mineral fertilizer application. Agronomy Journal. 2012; 104(6): 1558-1568.

20. Srivastava A, Dagbenonbakin GD, Gaiser T. Effect of fertilization on yam (*Dioscorea rotundata*) biomass production. Journal of Plant Nutrition. 2010; 33(7): 1056-1065.

21. Wu CL, Shen QR, Xia ZY, et al. Mechanisms for the increased utilization of fertilizer N under integrated use of inorganic and organic fertilizers in a winter wheat-rice rotation system. I. Fate of fertilizer 15N during winter wheat growing stages. Acta Pedologica Sinica. 2010; 47(5): 905-912.

22. Liu M, Linna C, Ma S, et al. Biochar combined with organic and inorganic fertilizers promoted the rapeseed nutrient uptake and improved the purple soil quality. Frontiers in Nutrition. 2022; 9: 997151.

23. Büyük G, Bayram C A, İnan M, et al. The effect of organic and inorganic fertilizers on plant nutrient content and agronomic performance of stevia. Journal of Plant Nutrition. 2022; 45(15): 2303-2314.

24. Qi Y, Jiang F, Zhou R, et al. Effects of reduced nitrogen with bio-organic fertilizer on soil properties, yield and quality of non-heading Chinese cabbage. Agronomy. 2021; 11(11): 2196.

25. Li K, Wang C, Li X, et al. Long-term effect of integrated fertilization on maize yield and soil fertility in a calcaric fluvisol. Archives of Agronomy and Soil Science. 2021; 67(10): 1400-1410.

26. Wang J, Zhang X, Yuan M, et al. Effects of Partial Replacement of Nitrogen Fertilizer with Organic Fertilizer on Rice Growth, Nitrogen Utilization Efficiency and Soil Properties in the Yangtze River Basin. Life. 2023; 13(3): 624.

27. Raza S T, Wu J, Ali Z, et al. Differential effects of organic amendments on maize biomass and nutrient availability in upland calcareous soil. Atmosphere. 2021; 12(8): 1034.

28. Moe K, Htwe AZ, Dien DC, et al. Effects of organic fertilizer applied using the estimated mineralizable nitrogen method on nitrogen uptake, growth characteristics, yield, and yield components of Genkitsukushi rice (*Oryza sativa*). Journal of Plant Nutrition. 2020; 43(10): 1400-1417.

29. Xiao X, Li J, Lyu J, et al. Chemical fertilizer reduction combined with bio-organic fertilizers increases cauliflower yield via regulation of soil biochemical properties and bacterial communities in Northwest China. Frontiers in Microbiology. 2022; 13: 922149.

30. Pouya N, Hgaza VK, Kiba DI, et al. Water yam (*Dioscorea alata* L.) growth and tuber yield as affected by rotation and fertilization regimes across an environmental gradient in West Africa. Agronomy. 2022; 12(4): 792.

31. Li FF, Zhu HY, Duan JS, et al. Effect of combined application of inorganic and organic fertilizer on wheat yield and fertilizer use efficiency in wheat following rice. Journal of Triticum Crops. 2018; 38(5): 593-599. (in Chinese)

32. Ma TT, Cai F, Ding CY, et al. Biological effects of special bio-organic fertilizer on yam. Journal of Nanjing Agricultural University. 2013; 36(4): 83-90. (in Chinese)

 Click here to access/download;Figure;Fig 2.docx

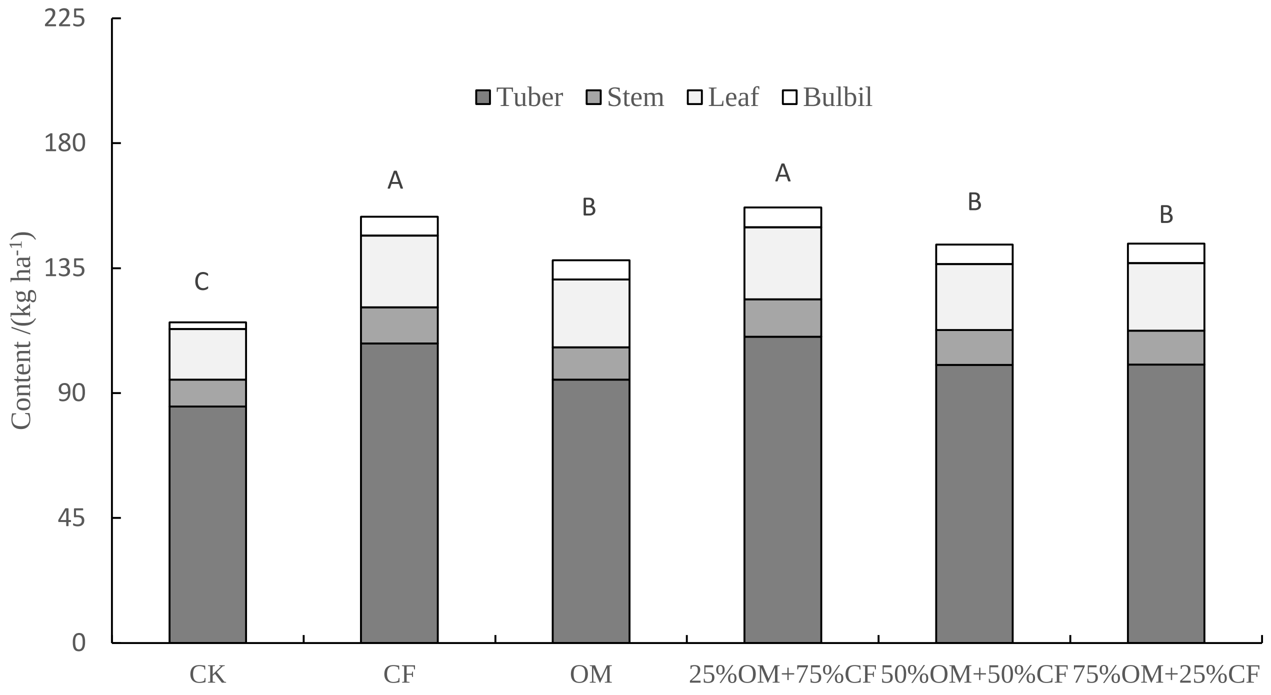

Note: Different letters indicate significant difference at $P < 0.05$ level.

Fig.2 Effect of partial substitution of chemical fertilizer with organic fertilizer on nitrogen accumulation in yam

 Click here to access/download;Figure;Fig 1.docx

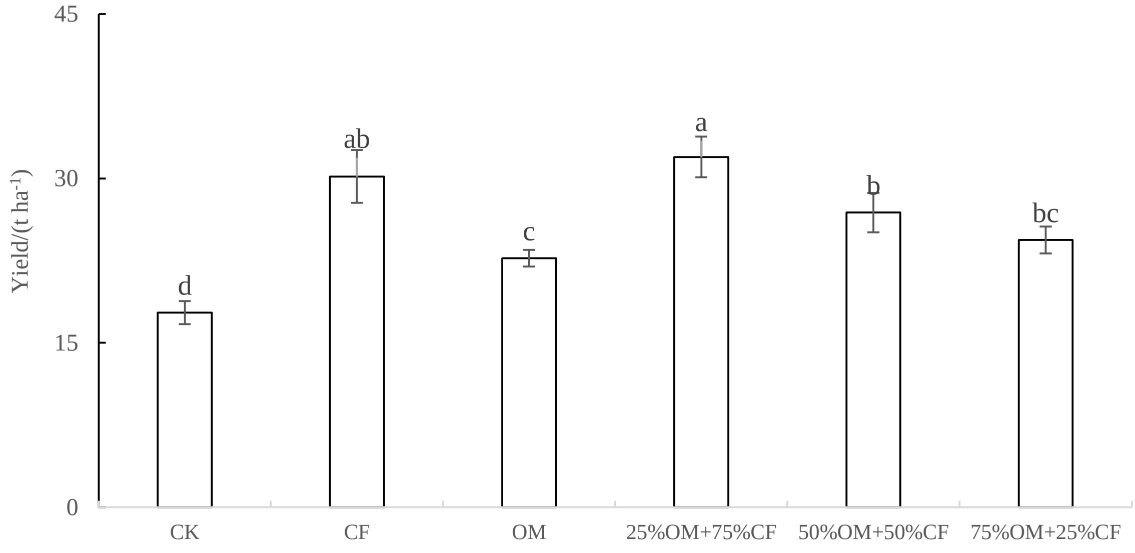

Note: Different letters indicate significant difference at $P < 0.05$ level.

Fig 1 Effect of partial substitution of chemical fertilizer with organic fertilizer on yam yield

Click here to access/download

Supporting Information - Compressed/ZIP File Archive

Supporting Information.rar

Revised Manuscript with Track Changes

Partial Substitution of Chemical Fertilizer with Organic Fertilizer Increases Yield, Quality and Nitrogen Utilization of *Dioscorea polystachya*

Chao Sun[1], Hua Zheng[2], Qing Zhao[2], Yuxi Liu[3], Hai Liu[1*]

[1]School of Management Science and Engineering, Guizhou University of Finance and Economics, Guiyang, Guizhou, China

[2]Guizhou Tobacco Company, China National Tobacco Corporation, Guiyang, Guizhou, China

[3]Academic Affairs Office, Guizhou University of Finance and Economics, Guiyang, Guizhou, China

[4]School of Literature, Guizhou University of Finance and Economics, Guiyang 550025, China)

* Corresponding author

E-mail: liuhai200321@163.com

Abstract

This field experiment aimed to investigate the effects of different ratios of organic and inorganic fertilizers with equal nitrogen application rates on the yield, quality, and nitrogen uptake efficiency of *Dioscorea polystachya* (yam). Six treatments were ~~established~~set, including a control without fertilizer (CK), sole application of chemical fertilizer (CF), sole application of organic fertilizer (OM), 25% organic fertilizer + 75% chemical fertilizer (25%OM + 75%CF), ~~and~~ 50% organic fertilizer + 50% chemical fertilizer (50%OM + 50%CF), and 75% organic fertilizer + 25% chemical fertilizer (75%OM + 25%CF). The experiment followed a randomized complete block design with three replications. Various yield parameters, morphology, quality indicators, and nitrogen utilization were analyzed to assess the differences among treatments. The results indicated that all fertilizer treatments significantly increased the yield, morphology, quality indicators, and nitrogen utilization efficiency compared to the control. 25%OM + 75%CF achieved the highest yield of 31.96 t hm^{-2}, which was not significantly different from CF (30.18 t hm^{-2}). Although there were no significant differences in tuber length and marketable yield among the fertilizer treatments, 25%OM + 75%CF exhibited the highest values at 69.23 cm and 75.86%, respectively, representing a 3.14% and 1.57% increase compared to CF. Tuber thickness and fresh weight of 25%OM + 75%CF showed no significant differences from CF, while OM and 50%OM+50%CF exhibited varying degrees of reduction compared to CF. The total sugar, starch, crude protein, total amino acid, and ash contents of *D. polystachya* significantly increased in all fertilizer treatments compared to the control (except for ash content between CK and OM). When compared to CF, the substitution of different ratios of organic fertilizer for chemical fertilizer increased the total sugar, starch, crude protein, total amino acid, and ash contents to varying degrees. The treatment with 25%OM+75%CF exhibited the highest increases of 6.31%, 3.78%, 18.40%, 29.70%, and 10%, respectively. Nitrogen content in different plant parts followed the sequence of tuber > leaves > stems > aerial stem, with the highest nitrogen accumulation observed in 25%OM + 75%CF treatment. Nitrogen harvest index did not show significant differences among

treatments, fluctuating between 0.69 and 0.74. The nitrogen apparent utilization efficiency was highest in 25%OM + 75%CF (9.89%), followed by CF (9.09%), both significantly higher than OM (5.32%) and 50%OM + 50%CF (6.69%). The nitrogen agronomic efficiency varied significantly among treatments, with 25%OM + 75%CF (33.93 kg kg^{-1}) being the highest, followed by CF (29.68 kg kg^{-1}), 50%OM + 50%CF (21.82 kg kg^{-1}), and OM (11.85 kg kg^{-1}). Nitrogen partial factor productivity was highest in 25%OM + 75%CF treatment (76.37 kg kg^{-1}), followed by CF (72.11 kg kg^{-1}), both significantly higher than 50%OM + 50%CF (64.25 kg kg^{-1}) and OM (54.29 kg kg^{-1}), with OM exhibiting significantly lower values compared to other treatments. In conclusion, the combined application of organic and inorganic fertilizers can effectively enhance the yield, quality, and nitrogen utilization efficiency of *D. polystachya*. Particularly, the treatment with 25% organic fertilizer and 75% chemical fertilizer showed the most promising results.

Key words: *Dioscorea polystachya*; partial substitution of chemical fertilizer with organic fertilizer; yield; quality; nitrogen utilization

Introduction

Yam (*Dioscorea polystachya*) is a perennial herbal plant that holds significant economic value as an important crop in China [1]. It is highly regarded for its nutritional and medicinal properties, serving as a crucial source of income for farmers [2]. However, the extensive growth cycle and high nutrient requirements of yam necessitate substantial fertilizer application. Unfortunately, in the pursuit of higher yields, farmers often resort to excessive use of chemical fertilizers, resulting in increased production costs and detrimental environmental effects [3, 4]. Research has revealed that an excessive proportion of unused fertilizers remains in the soil, leading to issues such as soil compaction and salinization [5]. Additionally, these unused fertilizers can enter the atmosphere and water bodies through various pathways like ammonia volatilization, nitrification, denitrification, surface runoff, and leaching. As a result, this contributes to worsening atmospheric pollution and eutrophication of water bodies [6, 7]. Consequently, it becomes imperative to explore comprehensive agricultural development models and nutrient management strategies that effectively meet China's food demands while minimizing the strain on resources and the environment [8]. This approach is crucial for achieving sustainable and "green" agricultural development in China [9].

Researchers have found that the use of organic fertilizer alone can lead to a significant decrease in crop yield. In low-input conditions, the application of organic fertilizer alone resulted in a 24% to 54% reduction in crop yield compared to the application of chemical fertilizer alone or the combined application of organic and inorganic fertilizers [10]. Furthermore, a 6-year field trial in Sweden demonstrated that using manure as the sole fertilizer resulted in a 47% and 82% decrease in barley and potato yields, respectively [11]. Many studies have highlighted the benefits of combining organic and chemical fertilizers, as this approach can boost crop yield and enhance soil quality. This

combined application strategy holds great potential for increasing agricultural production, improving efficiency, and reducing fertilizer usage in China.

In Guizhou province, the predominant practice has been the reliance on chemical fertilizers, with limited use of organic fertilizers. ~~To address this issue, this experiment aimed to determine the optimal ratio of organic fertilizer substitution for chemical fertilizer in the yam cultivation area of Guizhou.~~ The study assessed the effects of different proportions of organic fertilizer as a replacement for chemical fertilizer on yam yield, quality, morphology, and nitrogen utilization efficiency. The findings of this study aim to provide valuable guidance on reducing chemical fertilizer usage, promoting the appropriate application of organic fertilizer, and effectively enhancing yam yield in the Guizhou yam planting region.

Materials and methods

Overview of the test site

The experimental site was situated in the University Town of Huaxi District, Guiyang City, Guizhou Province, China (26° 22′N, 106° 38′E). This location falls within the hilly area of central Guizhou Province, characterized by an average altitude of 1030 m. The region experiences a subtropical monsoon climate, with an average annual temperature of 15.3℃ and approximately 1350 hours of sunshine per year. The frost-free period spans around 270 days, and annual rainfall typically ranges between 1100-1200 mm.

The experimental yellow soil (Eutric Regosol, UAO Soil Taxonomic System) ~~type~~was sampled from 0～20 cm soil layer prior to the experiment and then measured (analysis methods listed below). The soil derived from Triassic limestone and sand shale deposits, and had a sandy loam texture with pH 5.74 and contained 15.41 g/kg of organic matter (OM), 0.82 g/kg of total N (TN), 0.41 g/kg of total P (TP), 11.89 g/kg of total K (TK), 75.18 mg/kg of 1N NaOH-hydrolyzed N (AN), 16.8 mg/kg of Olsen P (AP), and 109.6 mg/kg of 1N CH_3COONH_4-exchangeable K (AK).

Test material

1) Yam variety: The test variety used in this study was Anshun Yam, a locally adapted variety obtained from the cultivation of wild yam plants in the region. This variety has undergone long-term domestication and natural selection. Seeds of Anshun Yam were acquired from Huaxi District Seed Company, located in Guiyang City, Guizhou Province, China.

2) Fertilizers: The organic ~~-~~fertilizer~~s~~ vermicompost, obtained by digesting decomposed cow dung with earthworms, ~~which~~ had a nutrient composition of N-P_2O_5-K_2O=2.01-2.41-1.03 and an organic matter content of 36%. The vermicompost was purchased from Guizhou Jilong Ecological Technology Co., LTD. In addition, synthetic fertilizers were used, including urea (N 46%), compound

Formatted: Font: (Default) Times New Roman

fertilizer (N-P_2O_5-K_2O =15-15-15), superphosphate (P_2O_5 16%), potassium sulfate (K_2O 50%), and diammonium hydrogen phosphate (N 18%, P_2O_5 46%). All of these synthetic fertilizers were procured from a local agricultural materials company.

Experimental design

The experiment was conducted over a two-year period, from 2020 to 2021, to evaluate the effects of different treatments on yam cultivation. Six treatments were established:

1) Control (CK): No fertilization was applied;

2) Chemical fertilizer (CF): Sole application of conventional chemical fertilizer;

3) Organic fertilizer (OM): Sole application of organic fertilizer;

4) 25%OM+75%CF: 25% organic fertilizer + 75% chemical fertilizer;

5) 50%OM+50%CF: 50% organic fertilizer + 50% chemical fertilizer~~.~~;

6) 75%OM+25%CF: 75% organic fertilizer + 25% chemical fertilizer.

The experiment was design in a randomized complete block design with three replicates. The experimental plot size was 7.5 m × 3.6 m. Each plot consisted of six rows, with a row spacing of 30 cm × 60 cm. A total of 180 yam plants (equivalent to 55 560 plants/hm^2) were planted in each plot, with a 0.3 m walkway separating the plots. Protective rows were established around the experimental plots to minimize edge effects.

In both 2020 and 2021, ~~the sowing of~~ yam setts were ~~seeds~~plant ~~took place~~ed on March 12 using the ~~branch~~ trench ~~sowing~~planting method ~~(. The~~ trench~~es were dug to a~~ depth ~~of~~ 15 cm). Prior to planting, a basal application of fertilizers was conducted, followed by ridging after planting (ridge height 15 cm). Staking was done before sprouting, and then trailing operations were continuously conducted by trailing newly sprouted yam vines to the stakes. Top dressing was applied approximately two months after planted. The field management practices were in accordance with local high-quality cultivation technical regulations. All fertilizing treatments were of the same application rates of N 418.5 kg/ha, P_2O_5 544.5 kg/ha, and K_2O 712.5 kg/ha. CF was the local conventional fertilizer treatment, with 2250 kg/ha compound fertilizer as base fertilizer, 750.0 kg/ha potassium sulfate plus 450.0 kg/ha ammonium dihydrogen phosphate as topdressing fertilizer. For the treatments combining organic and inorganic fertilizers, the organic fertilizer was applied in terms of nitrogen content, and any deficiency was supplemented with urea, calcium superphosphate, and potassium sulfate. Organic fertilizer and calcium superphosphate were exclusively used for basal application, while 60% of urea, potassium sulfate, and ammonium dihydrogen phosphate were used for basal application, and the remaining 40% was applied as topdressing. The fertilizer type and application amount were presented in Table 1.

Formatted: Font: (Default) Times New Roman

Formatted: Font: Times New Roman

Formatted: Font: Times New Roman

Table 1. Fertilization schemes ~~for~~of different ~~yam treated with different~~ treatments (kg/ha)

Treatments	Base fertilizer						Topdressing fertilizer		
	Compound fertilizer	Urea	Calcium superphosphate	Potassium sulfate	Ammonium dihydrogen phosphate	Organic fertilizer	Urea	Potassium sulfate	Ammonium dihydrogen phosphate
CK	0	0	0	0	0	0	0	0	0
CF	2250	0	0	0	0	0	0	750.0	450.0
OM	0	0	267.2	597.6	0	20820	0	398.4	0
25%OM+75%CF	0	409.4	2619	790.7	0	5205	273.0	527.1	0
50%OM+50%CF	0	273.0	1835	726.3	0	10410	182.0	484.2	0
75%OM+25%CF	0	136.5	1051	662.0	0	15615	91.05	441.3	0

Measuring items and methods

The yam was harvested during Nov. 21- Nov. 30, plot yield was weighed individually, soil (0～20 cm in depth) and yam tuber samples were collected through five-point sampling method in each plot separately. The measuring items and methods were listed below.

Soil properties: The soil samples collected from each plot were air-dried for determining the following indicators. The soil pH (soil:water =1:10) was measured with a pH metre (FE20, Mettler Toledo Co., Ltd., Shanghai, China), and OM was detected by the $K_2Cr_2O_7$ oxidation method. After digested with H_2SO_4-H_2O_2, the soil TN, TP, and TK in the digests were respectively determined by methods of Kjeldahl distillation, molybdenum blue colorimetry and flame photospectrometry [12]. For AN determination, the soil samples were evenly mixed with $FeSO_4{\cdot}7H_2O$-Ag_2SO_4 and added with 1 N NaOH solution (soil: solution = 1:5, weight: volume), after incubated at 40 °C for 24 h, HCl titration was employed to determine NH3 collected in 2% H_3BO_3 solution. 0.5 M $NaHCO_3$ (soil:water =1:10) was prepared for AP extraction, which was then measured using the colorimetric molybdenum-blue method. AK was extracted with 1 M NH4Ac and determined with a flame photometer (Shanghai Analysis Instrument Co., Ltd., China).

Yam yield: After sampling we harvested yam tubers per plot separately. The average yield of three replicate plots of each treatment was weighed and calculated to obtain the overall yield.

Tuber morphology: Ten random yam plants were selected from each plot to measure tuber morphology indices, including tuber length and tuber thickness.

Yam quality: Fresh and intact yam tubers of medium length were chosen from each plot to determine quality indices. The total ash content, soluble sugar content, starch content, and soluble protein content were determined respectively by the colorimetric analysis method [13].

Nutrient content: For nutrient analysis, five randomly selected yam plants were collected from

each plot. The stems, leaves, rhizomes, and tubers were weighed individually. The green parts were heated at 105℃ for 30 minutes to deactivate enzymes, and then dried at 65℃ to constant weight. The dry weight was recorded, and the samples were crushed for further analysis. After a deboiling process using concentrated sulfuric acid and hydrogen peroxide, the total nitrogen content (using the Kjeldahl method), total phosphorus content (using the vanadium-molybdenum yellow colorimetric method), and total potassium content (using a flame photometer) were determined for each plant part [14].

Relevant calculation formulas:

Plant nitrogen accumulation (kg hm^{-2}) = dry matter mass per plant × number of plants per hectare × nitrogen content of plants.

Nitrogen harvest index (NHI) = nitrogen accumulation of tuber / nitrogen accumulation of the whole plant.

Apparent nitrogen utilization ratio (%) = (nitrogen accumulation of plants treated with N fertilization－nitrogen accumulation of plants treated without N fertilization) / N fertilization amount × 100.

Nitrogen agronomic efficiency (kg/kg) = (yield of plants treated with N fertilization － yield of plants treated without N fertilization) / amount of nitrogen applied.

Nitrogen partial productivity (kg/kg) = yield / amount of nitrogen applied.

Data processing and analysis

All data were analyzed using one-way ANOVA in SAS 9.4 software (SAS Institute Inc., Cary, NC, USA). The analysis included the determination of yam yield, morphological indicators, total sugar, starch, crude protein, total amino acid, moisture, and ash content, as well as N accumulation in tuber, stem, leaf, and bulbil, and N use efficiency. Correlations between all above indicators in different treatments were also analyzed. The least significant difference (LSD) was employed for multiple comparisons between treatment means. The mean difference is considered significant when $P < 0.05$.

Results and analysis

Yield

As shown in Figure Fig 1, compared to the control treatment (CK), all fertilization treatments significantly increased yam yield ($P < 0.05$). The effect of different treatment varied. Among them, 25%OM + 75%CF had the highest yield, reaching 31.96 t hma^{-21}, which was significantly higher than OM (22.72 t hmha^{-21}) and, 50%OM + 50%CF (26.89 t hmha^{-21}) and 75%OM + 25%CF (24.38 t ha^{-1}), but showed no significant difference from CF (30.18 t hmha^{-21}). The yield increase achieved by OM was the weakest, significantly lower than that of other fertilizer treatments.

Morphological indexes

As shown in Table 2, fertilization significantly improved the morphological indexes of yam, although the effects varied among different fertilization treatments. 25%OM+75%CF resulted in the highest values for tuber length, tuber diameter, tuber fresh weight, and commercial rate, which were 69.23 cm, 5.51 cm, 1.15 kg, and 75.86%, respectively. There were no significant differences in tuber length and commodity rate among the different treatments, with values ranging from 63.48 cm to 69.23 cm and from 74.12% to 75.86%, respectively. However, the tuber diameter of OM was significantly lower than that of CF and 25%OM+75%CF (5.48 cm and 5.51 cm, respectively). The tuber fresh weight showed significant differences among different treatments, except for the non-significant difference of CF (1.11 kg) with 25%OM+75%CF (1.15 kg), as well as that of 75%OM+25%CF (0.82 kg) with OM (0.80 kg) and 50%OM+50%CF (0.91 kg). Significant differences were observed among the other treatments (*P* < 0.05).

Table 2. Effect of partial substitution of chemical fertilizer with organic fertilizer on morphological indicators of yam

Treatment	Tuber length /cm	Tuber thickness /cm	Tuber fresh weight /kg	Commodity rate /%
CK	56.78± 1.08c	4.66± 0.13c	0.68± 0.07d	60.87± 1.03b
CF	67.12± 1.35a	5.48± 0.14a	1.11± 0.12a	74.69± 1.77a
OM	63.48±0.81 ab	4.87± 0.07b	0.80± 0.06c	75.16± 2.38a
25%OM+75%CF	69.23± 2.17a	5.51± 0.26a	1.15± 0.13a	75.86± 2.65a
50%OM+50%CF	65.31±1.79 ab	5.03± 0.11ab	0.91± 0.05b	74.12± 1.14a
75%OM+25%CF	64.63±1.21 ab	4.92±0.09 ab	0.82±0.08 bc	74.31±0.82 a

Note: Different letters in the same column indicate significant level of difference P < 0.05

Quality

Application of fertilizer has a significant impact on the quality of yam. Fertilization led to a significant increase in the contents of total sugar, starch, crude protein, total amino acid, and ash in yam (except for OM), while significantly reducing the water content of yam (*P* < 0.05). The contents of total sugar, starch, water, and ash in each fertilizer treatment varied within the ranges of 12.21%～12.98%, 15.62%～16.21%, 75.43%～76.95%, and 0.66%～0.77%, respectively. However, no significant differences were observed among the different treatments. Compared to CF, partial substitution of chemical fertilizer with organic fertilizer significantly increased the contents of crude protein and total amino acid in yam, with the treatment of 25%OM+75%CF showing the highest increases at 18.40% and 29.70%, respectively (Table 3).

Table 3. Effect of partial substitution of chemical fertilizer with organic fertilizer on yam quality

Treatment	Total sugar /%	Starch /%	Crude protein /%	Total amino acids /%	Moisture /%	Ash /%
CK	10.43± 0.28b	14.01± 0.08b	2.81± 0.07c	1.74±0.03 c	81.23± 1.68a	0.61± 0.02b

CF	12.21± 0.31a	15.62± 0.11a	3.37± 0.11b	2.02± 0.05b	76.95± 1.94b	0.70± 0.01a
OM	12.69± 0.24a	15.66± 0.18a	3.91± 0.16a	2.59±0.06 a	75.18± 1.79b	0.66± 0.04ab
25%OM+75%CF	12.98± 0.33a	16.21± 0.13a	3.99± 0.15a	2.62±0.09 a	75.44± 2.14b	0.77±0.03 a
50%OM+50%CF	12.77±0.29 a	15.78± 0.17a	3.86± 0.19a	2.55± 0.02a	76.25± 1.81b	0.71± 0.05a
75%OM+25%CF	12.75±0.17 a	15.71± 0.14a	3.87± 0.12a	2.52± 0.04a	75.43± 1.26b	0.67± 0.04ab

Note: Different letters in the same column indicate significant difference at *P* < 0.05 level.

Nitrogen accumulation

The distribution of nitrogen accumulation in different parts of yam follows the pattern of tuber > leaf > stem > bulbil, with proportions ranging from 68.81% to 73.76%, 15.74% to 17.73%, 8.35% to 8.72%, and 2.16% to 4.95%, respectively (Fig. 2). In terms of total nitrogen accumulation, fertilization significantly increased the nitrogen accumulation in yam (P < 0.05). 25%OM+75%CF had the highest nitrogen accumulation at 156.91 kg hmha^{-21}, followed by CF (153.56 kg hmha^{-21}), with no significant difference between them. However, both were significantly higher than that of OM (137.78 kg hmha^{-21}), 50%OM+50%CF (143.49 kg ha^{-1}), and 5075%OM+5025%CF (143.49 78 kg hmha^{-21}).

Nitrogen utilization

As shown in Table 4, the nitrogen harvest index of the control treatment (CK) was the highest, reaching 0.74. However, there were no significant differences between CK and other treatments, and the nitrogen harvest index (NHI) ranged between 0.69 and 0.74. 25%OM+75%CF had the highest nitrogen apparent utilization rate at 9.89%, followed by CF(9.09%). Both of these values were significantly higher than 75%OM+25%CF (6.76%), 50%OM+50%CF (6.69%) and OM (5.32%). Significant differences were observed in nitrogen agronomic efficiency among the different treatments. 25%OM+75%CF had the highest nitrogen agronomic efficiency at 33.93 kg kg^{-1}, followed by CF (29.68 kg kg^{-1}). 5075%OM+5025%CF and OM had the lowest nitrogen agronomic efficiency of 21.8215.82 kg kg^{-1} and 11.85 kg kg^{-1}, respectively. The nitrogen partial productivity was the highest for 25%OM+75%CF at 76.37 kg kg^{-1}, followed by CF (72.11 kg kg^{-1}). There were no significant differences between these two treatments, but both were significantly higher than 50%OM+50%CF (64.25 kg kg^{-1}), 75%OM+25%CF (58.26 kg kg^{-1}) and OM (54.29 kg kg^{-1}).

Table 4. Effect of organic and chemical fertilizer application on nitrogen use efficiency of yam(mean ± standard deviation)

Treatment	NHI	Apparent utilization rate /%	Agronomic efficiency /(kg kg^{-1})	Partial productivity /(kg kg^{-1})
CK	0.74±0.01 a	—	—	—
CF	0.70±0.03 a	9.09±0.44 a	29.68±1.79 b	72.11±2.44 a
OM	0.69±0.02 a	5.32±0.51 b	11.85±0.46 de	54.29±2.19 c
25%OM+75%CF	0.70±0.01 a	9.89±0.76 a	33.93±1.35 a	76.37±3.76 a
50%OM+50%CF	0.70±0.02 a	6.69±0.48 b	21.82±1.08 c	64.25±5.48 b
75%OM+25%CF	0.70±0.01 a	6.76±0.44 b	15.82±1.37 d	58.26±4.47 c

Note: Different letters in the same column indicate significant difference at $P < 0.05$ level.

Discussion

It is worth underscoring that achieving the right balance between organic and chemical fertilizers is pivotal in optimizing nitrogen absorption, crop growth, and yield. This equilibrium not only enhances the understanding of nutrient dynamics in yam cultivation but also offers valuable insights for sustainable and high-yield agricultural practices in a broader context. In comparison to the conventional fertilizer treatment (CF), the substitution of 25% of chemical fertilizer with organic fertilizer resulted in a significant increase in yam yield. However, when organic fertilizer replaced 50% of the chemical fertilizer, there was a notable reduction in yield [15]. Prior research has suggested that an exclusive reliance on organic fertilizer as the sole nitrogen source can lead to yield decreases [16]. Consequently, it is imperative to consider the proportional blend of organic and chemical fertilizers. Under conditions of equal nitrogen supply, replacing 50% of the fertilizer's nitrogen with organic fertilizer can lead to a slower release of nutrients and reduced ammonium and nitrate nitrogen content in the soil. As yam's nitrogen requirements intensify during the later growth stages, these demands cannot be adequately met, ultimately resulting in a substantial decline in yield. This observation aligns with prior studies [17]. Our two-year fertilization experiment illustrates the viability of replacing a portion of chemical fertilizer with organic fertilizer in yam cultivation in Guizhou Province. We conclude that maintaining a 25% ratio of organic fertilizer nitrogen to fertilizer nitrogen preserves high tuber length, crude and fresh weight, and commercial rate in yam crops, ensuring a consistent and substantial yield.

Applying nitrogen correctly has been demonstrated to be highly effective in enhancing nitrogen absorption intensity and distribution rates in yam grains. The combined application of organic and chemical fertilizers has proven to increase nitrogen accumulation in yam plants and notably elevate the nitrogen content in the stems [18-19]. Our study revealed that the nitrogen accumulation and distribution ratio in yam stems significantly surpassed that of other plant parts, consistent with earlier research findings [20]. Under conditions of isonitrogen fertilization, replacing 25% of chemical fertilizers with organic fertilizer resulted in significantly greater total nitrogen accumulation throughout the entire plant and in the yam tubers compared to the conventional fertilizer treatment. Wu et al. [21], utilizing 'N labeling technology, also illustrated that combining organic and inorganic fertilizers could enhance nitrogen accumulation in grains within both vegetative and reproductive plant organs of winter wheat, surpassing the effects of single applications of organic or inorganic fertilizers. Optimal accumulation and distribution of crop dry matter play a pivotal role in achieving high-quality and high-yield yam production. The combined application of organic and chemical fertilizers proves beneficial in optimizing water and nutrient conditions, promoting overall dry matter accumulation throughout the crop's growth cycle, and facilitating the translocation of dry matter to the stems [22-23].

Nonetheless, as the proportion of organic fertilizer nitrogen relative to fertilizer nitrogen increases, both the total nitrogen accumulation in plants and grains gradually decreases, in accordance with prior studies [24-27]. Moreover, it has been observed that with the growing proportion of organic fertilizer substituting chemical fertilizer, there is an increase in organic matter content. However, the mineralization process of organic matter is relatively slow, leading to a delayed release of nutrients. Consequently, the nitrogen mineralized from organic fertilizer may prove insufficient to meet the plant's nitrogen uptake requirements, resulting in inadequate absorption of available nitrogen and ultimately lower nitrogen accumulation in the stems [28-29].

The challenge of low nitrogen utilization rates remains a prominent issue in Chinese crop production. Various indicators, including nitrogen harvest index, nitrogen apparent utilization rate, agronomic efficiency, and partial productivity, serve to gauge nitrogen efficiency. Within the scope of this study, significant differences were observed concerning both agronomic efficiency and partial productivity of nitrogen fertilizer. These findings signify an imbalance in the fertilization system in the region, stemming from the application of nitrogen, phosphorus, and potassium or possibly excessive fertilizer use. Consequently, optimizing the fertilizer composition becomes a necessity. Research has indicated that, under specific nitrogen application levels, reducing chemical fertilizer application while increasing organic fertilizer usage can substantially enhance yam's fertilizer utilization efficiency. It is crucial to emphasize that the optimization should target the organic fertilizer-to-fertilizer nitrogen ratio rather than merely seeking a higher ratio [30]. Li et al. [31] discovered that the peak nitrogen fertilizer agronomic use efficiency in wheat was attained when organic fertilizer replaced 20% of the chemical nitrogen fertilizer. In our study, we observed a similar pattern, with nitrogen fertilizer efficiency initially increasing and then decreasing as the proportion of organic fertilizer nitrogen replacing chemical fertilizer nitrogen rose. The highest nitrogen use efficiency for yams was achieved when organic fertilizer nitrogen replaced 25% of chemical fertilizer nitrogen. This outcome aligns with previous research results [32]. These findings underscore the importance of a nuanced approach to nitrogen management in yam cultivation, seeking the optimal balance between organic and chemical fertilizers to maximize nitrogen utilization efficiency and overall crop productivity. The results of this study provide valuable insights for sustainable agricultural practices and contribute to the broader understanding of nutrient management in crop production.

Conclusion

Partial substituting chemical fertilizers with organic fertilizers performed differently as the substitution ratio varied in increasing yam yield, quality and nitrogen utilization of *D. polystachya*. Replacing 25% chemical fertilizers with organic fertilizer had comparable or even better performance than totally applying chemical fertilizers, specifically, the yam yield, tuber length, thickness, fresh weight, commodity rate, contents of total sugar, starch, moisture and ash, nitrogen accumulation

amount, as well as nigrogen harvest index, apparent utilization rate and partial productivity didn't vary significantly between CF and 25%OM+75%CF treatments, while the latter one significantly increased the contents of crude protein and total amino acids, and the agronomic efficiency. At the current stage, taking into the economic benefits for farmers, it is not practical to solely use organic fertilizers without applying chemical fertilizers.

Acknowledgements

Many thanks to Dr. Jianguo Huang at Southwest University and anonymous reviewers for their valuable comments on this manuscript.

Author Contributions

Data curation: Chao Sun, Yuxi Liu.

Software: Qing Zhao.

Writing – original draft: Chao Sun, Hai Liu.

Writing – review & editing: Hai Liu.

References

1. Fan D, Zhong H, Hu B, et al. Agro-ecological suitability assessment of Chinese Medicinal Yam under future climate change. Environmental Geochemistry and Health. 2020; 42: 987-1000.

2. Li Y, Ji S, Xu T, et al. Chinese yam (*Dioscorea*): Nutritional value, beneficial effects, and food and pharmaceutical applications. Trends in Food Science & Technology. 2023; 134: 29-40.

3. He R, Shao C, Shi R, et al. Development trend and driving factors of agricultural chemical fertilizer efficiency in China. Sustainability. 2020, 12(11): 4607. https://doi.org/10.3390/su12114607.

4. Dimkpa CO, Fugice J, Singh U, et al. Development of fertilizers for enhanced nitrogen use efficiency–Trends and perspectives. Science of the Total Environment. 2020; 731: 139113.

5. Stavi I, Thevs N, Priori S. Soil salinity and sodicity in drylands: A review of causes, effects, monitoring, and restoration measures. Frontiers in Environmental Science. 2021: 330.

6. Brentrup F, Küsters J, Kuhlmann H, et al. Environmental impact assessment of agricultural production systems using the life cycle assessment methodology: I. Theoretical concept of a LCA method tailored to crop production. European Journal of Agronomy. 2004; 20(3): 247-264.

7. Sun B, Zhang L, Yang L, et al. Agricultural non-point source pollution in China: causes and mitigation measures. Ambio. 2012; 41: 370-379.

8. Chivenge P, Zingore S, Ezui K S, et al. Progress in research on site-specific nutrient management for smallholder farmers in sub-Saharan Africa. Field Crops Research. 2022; 281: 108503.

9. Liu P, He J, Li H, et al. Effect of straw retention on crop yield, soil properties, water use efficiency and greenhouse gas emission in China: A meta-analysis. International Journal of Plant Production. 2019; 13: 347-367.

10. Vanlauwe B, Kihara J, Chivenge P, et al. Agronomic use efficiency of N fertilizer in maize-based systems in sub-Saharan Africa within the context of integrated soil fertility management. Plant and soil. 2011; 339: 35-50.

11. Torstensson G, Aronsson H, Bergström L. Nutrient use efficiencies and leaching of organic and conventional cropping systems in Sweden. Agronomy Journal. 2006; 98(3): 603-615.

12. Pansu M. Handbook of soil analysis. Heidelberg: Springer; 2006.

13. Leng F, Sun S, Jing Y, et al. A rapid and sensitive method for determination of trace amounts of glucose by anthrone-sulfuric acid method. Bulg Chem Commun. 2016; 48(1): 109-113.

14. Motsara MR. Guide to laboratory establishment for plant nutrient analysis. Jodhpur: Scientific Publishers; 2015.

~~14.~~15. Suja G, Sreekumar J. Implications of organic management on yield, tuber quality and soil health in yams in the humid tropics. International Journal of Plant Production. 2014; 8(3): 291-310.

~~15.~~16. Geng Y, Cao G, Wang L, et al. Effects of equal chemical fertilizer substitutions with organic manure on yield, dry matter, and nitrogen uptake of spring maize and soil nitrogen distribution. PloS one, 2019; 14(7): e0219512.

~~16.~~17. Divito GA, Sadras VO. How do phosphorus, potassium and sulphur affect plant growth and biological nitrogen fixation in crop and pasture legumes? A meta-analysis. Field Crops Research. 2014; 156: 161-171.

18. KV H, Oberson A, Kiba I D, et al. The nitrogen nutrition of yam (*Dioscorea* spp). Journal of Plant Nutrition. 2020; 43(1): 64-78.

~~17.~~19. Hgaza VK, Diby LN, Oberson A, et al. Nitrogen use by yam as affected by mineral fertilizer application. Agronomy Journal. 2012; 104(6): 1558-1568.

~~18.~~20. Srivastava A, Dagbenonbakin GD, Gaiser T. Effect of fertilization on yam (*Dioscorea rotundata*) biomass production. Journal of Plant Nutrition. 2010; 33(7): 1056-1065.

~~19.~~21. Wu CL, Shen QR, Xia ZY, et al. Mechanisms for the increased utilization of fertilizer N under integrated use of inorganic and organic fertilizers in a winter wheat-rice rotation system. I. Fate of fertilizer 15N during winter wheat growing stages. Acta Pedologica Sinica. 2010; 47(5): 905-912.

~~20.~~22. Liu M, Linna C, Ma S, et al. Biochar combined with organic and inorganic fertilizers promoted the rapeseed nutrient uptake and improved the purple soil quality. Frontiers in Nutrition. 2022; 9:

997151.

~~21.~~23. Büyük G, Bayram C A, İnan M, et al. The effect of organic and inorganic fertilizers on plant nutrient content and agronomic performance of stevia. Journal of Plant Nutrition. 2022; 45(15): 2303-2314.

~~22.~~24. Qi Y, Jiang F, Zhou R, et al. Effects of reduced nitrogen with bio-organic fertilizer on soil properties, yield and quality of non-heading Chinese cabbage. Agronomy. 2021; 11(11): 2196.

~~23.~~25. Li K, Wang C, Li X, et al. Long-term effect of integrated fertilization on maize yield and soil fertility in a calcaric fluvisol. Archives of Agronomy and Soil Science. 2021; 67(10): 1400-1410.

~~24.~~26. Wang J, Zhang X, Yuan M, et al. Effects of Partial Replacement of Nitrogen Fertilizer with Organic Fertilizer on Rice Growth, Nitrogen Utilization Efficiency and Soil Properties in the Yangtze River Basin. Life. 2023; 13(3): 624.

~~25.~~27. Raza S T, Wu J, Ali Z, et al. Differential effects of organic amendments on maize biomass and nutrient availability in upland calcareous soil. Atmosphere. 2021; 12(8): 1034.

28. Moe K, Htwe AZ, Dien DC, et al. Effects of organic fertilizer applied using the estimated mineralizable nitrogen method on nitrogen uptake, growth characteristics, yield, and yield components of Genkitsukushi rice (*Oryza sativa*). Journal of Plant Nutrition. 2020; 43(10): 1400-1417.

~~26.~~29. Xiao X, Li J, Lyu J, et al. Chemical fertilizer reduction combined with bio-organic fertilizers increases cauliflower yield via regulation of soil biochemical properties and bacterial communities in Northwest China. Frontiers in Microbiology. 2022; 13: 922149.

~~27.~~30. Pouya N, Hgaza VK, Kiba DI, et al. Water yam (*Dioscorea alata* L.) growth and tuber yield as affected by rotation and fertilization regimes across an environmental gradient in West Africa. Agronomy. 2022; 12(4): 792.

~~28.~~31. Li FF, Zhu HY, Duan JS, et al. Effect of combined application of inorganic and organic fertilizer on wheat yield and fertilizer use efficiency in wheat following rice. Journal of Triticum Crops. 2018; 38(5): 593-599. (in Chinese)

~~29.~~32. Ma TT, Cai F, Ding CY, et al. Biological effects of special bio-organic fertilizer on yam. Journal of Nanjing Agricultural University. 2013; 36(4): 83-90. (in Chinese)

Dear editor,

Thank you very much for your positive comments on our manuscript entitled "Partial Substitution of Chemical Fertilizer with Organic Fertilizer Increases Yield, Quality and Nitrogen Utilization of Dioscorea polystachya" (PONE-D-23-21993). All the comments are valuable and helpful for improving our paper. We substantially revised our old version based on the reviewer's comments. Thereafter, the manuscript was revised and edited by a native speaker and we hope the revised manuscript can meet the requirements. In this thoroughly revised version, changes in our manuscript within the document were highlighted by using red-colored text. Point-by-point responses to the reviewers are included, and the line number based on the attached file named "Revised Manuscript with track changes".

We would like to express our great appreciation to you and reviewers for comments on our paper. Looking forward to hearing from you.

Thank you and best regards.

Reviewer #1:

L14 These are five treatments and not six.

Response: Thank you very much for your comments. Your suggestions are beneficial to improve our present manuscript and our further research. We added the sixth treatment 75% organic fertilizer + 25% chemical fertilizer, as well as the related data in the following figures and tables.

L74 Why aim and objective in the same paragraph? Please stick to one

Response: We have delete one of them.

L88 space figures and letters

Response: Revised.

L89 How did you get this information?

Response: We rewrote this paragraph, and placed the detailed analysis methods in L143-153.

L100 were all these fertilizers combined together and used as one??? if no, how and when did you apply each of these fertilizers???

Response: The specific application methods were rewrote and presented in L121-136.

L110 There should be six treatment in all. The study should also include higher level of OM and lower level of CF. ie

75%OM+25%CF: 75% organic fertilizer + 25% chemical fertilizer

Response: We have added the sixth treatment 75%OM+25%CF.

L121 there is a major difference between sowing and planting. when vegetative part of a plant is propagated it is referred to as planting while when the seed is used it is refereed to as sowing. i believe you used the vegetative part of the plant

Response: Thanks a lot for your suggestion, we have replaced the word ‘sowing’ with ‘planting’.

L122 which of the fertilizer???

Response: We rewrote this paragraph to clarify this question in L121-136.

L127 what did you mean by this???

Response: Treatment 2 is the local conventional high-quality cultivation, based on this, we set the application amounts of fertilizers. we have rewrote this paragraph to stated the fertilizer application stages and amounts in detail.

L143 This is not acceptable. Kindly state how soil samples were collected and analysed

Response: We have stated the soil samples collecting and analyzing methods in detail in L143-153.

L160 You need to reference the methods used

Response: Related reference is added.

L167 reference is missing

Response: Related reference is added.

L183 I cant find the correlation matrix/table in the text

Response: This sentence ‘Correlations between all above indicators in different treatments were also analyzed’ is deleted.

L248 Discussion should come before conclusion. This section is not well discussed, needs a lot of improvements

Reviewer #2:

L100 What was the input to obtain the vermicompost?

Response: Revised. The vermicompost was obtained by digesting decomposed cow dung with earthworms.

L116 Why only 3 repetitions? Considering the experimental design, the number of treatments and replicates, the degrees of freedom of error are few. A serious experiment considers at least 12 degrees of freedom from error.

Response: Thanks a lot for your suggestions, while in agricultural field experiments, three replicates are commonly applied, and is proved to be appropriate for field fertilizer experiments. This can be verified in numerous published papers.

L183 No correlation results are presented.

Response: Revised.

L226 Maintain uniformity in the literals. In lower case as in figure 1.

Response: Revised.

L248 Further discussion related to the characteristics and effects of the organic material applied is required.

Response: The discussion part is already rewrote.

L248 Practically only nitrogen is discussed, what about the other essential elements?

Response: In yam growth, the element nitrogen plays am important role, so we measured some nitrogen-related indicator in our paper. While P and K elements were not detected particularly in the manuscript, and thus cannot be discussed.

L255 Any organic fertilizer?

Response: Revised.

L255\262\369\372 In the list of references it appears as 2020

Response: Revised.
