# Supplementary material for: Partial substitution of chemical fertilizer by organic fertilizer increases yield, quality and nitrogen utilization of Dioscorea polystachya
Source: PLoS One. 2024 Apr 11;19(4):e0301108. doi: 10.1371/journal.pone.0301108 (PMC11008863; doi:10.1371/journal.pone.0301108)
Supplement: Attachment — Submitted filename: Response to Reviewers.docx [file pone.0301108.s004.docx]

Dear editor,

Thank you very much for your positive comments on our manuscript entitled “Partial Substitution of Chemical Fertilizer with Organic Fertilizer Increases Yield, Quality and Nitrogen Utilization of Dioscorea polystachya” (PONE-D-23-21993). All the comments are valuable and helpful for improving our paper. We substantially revised our old version based on the reviewer’s comments. Thereafter, the manuscript was revised and edited by a native speaker and we hope the revised manuscript can meet the requirements. In this thoroughly revised version, changes in our manuscript within the document were highlighted by using red-colored text. Point-by-point responses to the reviewers are included, and the line number based on the attached file named “Revised Manuscript with track changes”.

We would like to express our great appreciation to you and reviewers for comments on our paper. Looking forward to hearing from you.

Thank you and best regards.

**Reviewer #1:**

**L14**	These are five treatments and not six.

**Response:** Thank you very much for your comments. Your suggestions are beneficial to improve our present manuscript and our further research. We added the sixth treatment 75% organic fertilizer + 25% chemical fertilizer, as well as the related data in the following figures and tables.

**L74** Why aim and objective in the same paragraph? Please stick to one

**Response:** We have delete one of them.

**L88** space figures and letters

**Response:** Revised.

**L89** How did you get this information?

**Response:** We rewrote this paragraph, and placed the detailed analysis methods in L143-153.

**L100** were all these fertilizers combined together and used as one??? if no, how and when did you apply each of these fertilizers???

**Response:** The specific application methods were rewrote and presented in L121-136.

**L110** There should be six treatment in all. The study should also include higher level of OM and lower level of CF. ie

75%OM+25%CF: 75% organic fertilizer + 25% chemical fertilizer

**Response:** We have added the sixth treatment 75%OM+25%CF.

**L121** there is a major difference between sowing and planting. when vegetative part of a plant is propagated it is referred to as planting while when the seed is used it is refereed to as sowing. i believe you used the vegetative part of the plant

**Response:** Thanks a lot for your suggestion, we have replaced the word ‘sowing’ with ‘planting’.

**L122** which of the fertilizer???

**Response:** We rewrote this paragraph to clarify this question in L121-136.

**L127** what did you mean by this???

**Response:** Treatment 2 is the local conventional high-quality cultivation, based on this, we set the application amounts of fertilizers. we have rewrote this paragraph to stated the fertilizer application stages and amounts in detail.

**L143** This is not acceptable. Kindly state how soil samples were collected and analysed

**Response:** We have stated the soil samples collecting and analyzing methods in detail in L143-153.

**L160** You need to reference the methods used

**Response:** Related reference is added.

**L167** reference is missing

**Response:** Related reference is added.

**L183** I cant find the correlation matrix/table in the text

**Response:** This sentence ‘Correlations between all above indicators in different treatments were also analyzed’ is deleted.

**L248** Discussion should come before conclusion. This section is not well discussed, needs a lot of improvements

**Reviewer #2:**

**L100** What was the input to obtain the vermicompost?

**Response:** Revised. The vermicompost was obtained by digesting decomposed cow dung with earthworms.

**L116** Why only 3 repetitions? Considering the experimental design, the number of treatments and replicates, the degrees of freedom of error are few. A serious experiment considers at least 12 degrees of freedom from error.

**Response:** Thanks a lot for your suggestions, while in agricultural field experiments, three replicates are commonly applied, and is proved to be appropriate for field fertilizer experiments. This can be verified in numerous published papers.

**L183** No correlation results are presented.

**Response:** Revised.

**L226** Maintain uniformity in the literals. In lower case as in figure 1.

**Response:** Revised.

**L248** Further discussion related to the characteristics and effects of the organic material applied is required.

**Response:** The discussion part is already rewrote.

**L248** Practically only nitrogen is discussed, what about the other essential elements?

**Response:** In yam growth, the element nitrogen plays am important role, so we measured some nitrogen-related indicator in our paper. While P and K elements were not detected particularly in the manuscript, and thus cannot be discussed.

**L255** Any organic fertilizer?
**Response:** Revised.

**L255\262\369\372** In the list of references it appears as 2020
**Response:** Revised.
