# Supplementary material for: Partial substitution of chemical fertilizer by organic fertilizer increases yield, quality and nitrogen utilization of Dioscorea polystachya
Source: PLoS One. 2024 Apr 11;19(4):e0301108. doi: 10.1371/journal.pone.0301108 (PMC11008863; doi:10.1371/journal.pone.0301108)
Supplement: Attachment — Submitted filename: Response to Reviewers0113.docx [file pone.0301108.s006.docx]

Dear editor,

Thank you very much for your positive comments on our manuscript entitled “Partial Substitution of Chemical Fertilizer with Organic Fertilizer Increases Yield, Quality and Nitrogen Utilization of Dioscorea polystachya” (PONE-D-23-21993). All the comments are valuable and helpful for improving our paper. We substantially revised our old version based on the reviewer’s comments. Thereafter, the manuscript was revised and edited by a native speaker and we hope the revised manuscript can meet the requirements. In this thoroughly revised version, changes in our manuscript within the document were highlighted by using red-colored text. Point-by-point responses to the reviewers are included, and the line number based on the attached file named “Revised Manuscript with track changes”.

We would like to express our great appreciation to you and reviewers for comments on our paper. Looking forward to hearing from you.

Thank you and best regards.

**1.** Title

- replace "with organic fertilizer" by "by organic fertilizer.

**Response:** revised.

**2.** Abstract

- Please rephrase this sentence - "Although there were no significant differences in tuber length and marketable yield among the fertilizer treatments, 25%OM + 75% CF exhibited the highest values at 69.23 cm and 75.86%, respectively, representing a 3.14% and 1.57% increase compared to CF. If there is not significant differences the authors could not state increases compared to control.

**Response:** We have rephrased this sentence.

**3.** - Please rephrase the following sentences, as the infomration is repeated : The total sugar, starch, crude protein, total amino acid, and ash contents of D. polystachya significantly increased in all fertilizer treatments compared to the control (except for ash content between CK and OM). When compared to CF, the substitution of different ratios of organic fertilizer for chemical fertilizer increased the total sugar, starch, crude protein, total amino acid, and ash contents to varying degrees."

**Response:** Rephrased.

**4.** Line 137- The following sub-heading should be changed: " Measuring items and methods"? Not clear, please rephrase for clarity.

**Response:** Revised. Changed into “Methods of index determination”.

**5.** Line 177 - Please replace by "Data analysis"

**Response:** Changed into "Data analysis".

**6.** Discussion

- Data regarding yam's quality are not discussed. Please imporve the discussion section.

**Response:** We have added the yam quality discussion, and also improved the presentation.

**7.** The manuscript must be revised by a Native English speaker as it contains several grammatical faults.

**Response:** Revised.
