# Supplementary material for: Partial substitution of chemical fertilizer by organic fertilizer increases yield, quality and nitrogen utilization of Dioscorea polystachya
Source: PLoS One. 2024 Apr 11;19(4):e0301108. doi: 10.1371/journal.pone.0301108 (PMC11008863; doi:10.1371/journal.pone.0301108)
Supplement: Attachment — Submitted filename: Response to Reviewers.docx [file pone.0301108.s007.docx]

Dear editor,

Thank you very much for your positive comments on our manuscript entitled “Partial Substitution of Chemical Fertilizer with Organic Fertilizer Increases Yield, Quality and Nitrogen Utilization of Dioscorea polystachya” (PONE-D-23-21993). All the comments are valuable and helpful for improving our paper. We substantially revised our old version based on the reviewer’s comments. Thereafter, the manuscript was revised and edited by a native speaker and we hope the revised manuscript can meet the requirements. In this thoroughly revised version, changes in our manuscript within the document were highlighted by using red-colored text. Point-by-point responses to the reviewers are included, and the line number based on the attached file named “Revised Manuscript with track changes”.

We would like to express our great appreciation to you and reviewers for comments on our paper. Looking forward to hearing from you.

Thank you and best regards.

**1.**  The manuscript needs to be revised by a native English speaker. Several grammar faults and unclear sentences are found throughout the text.

**Response:** Thanks a lot for your suggestion. I have taken the necessary steps to enhance the linguistic quality of our manuscript. I engaged the assistance of a faculty member from the University of Florida, who provided valuable insights and refined the language throughout the paper.
